# Supplementary material for: Use, characteristics and influence of lay consultation networks on treatment-seeking decisions in slums of Nigeria: a cross-sectional survey
Source: BMJ Open. 2023 May 16;13(5):e065152. doi: 10.1136/bmjopen-2022-065152 (PMC10193090; doi:10.1136/bmjopen-2022-065152)
Supplement: Supplementary data [file bmjopen-2022-065152supp001.pdf]

Table 1: Survey instrument

		**Section One: Sociodemographic Characteristics of respondents**<br><br>I would like to begin by asking you some questions about yourself.	
1	Age	How old were you on your last birthday?	(____)
2	Gender	What is your sex?	Male………...1<br>Female……...2
3	Digital Communication	a. Do you carry a mobile phone day-to-day?	Yes………………1<br>No…….……….....2<br>Don't know………3
		b. How many days in the week do you have access to internet/Wi-Fi (for example, Facebook, email) using digital communication device (like smart phone, laptop, tablet)?	Everyday…………..1<br>Almost every day (more than 21 days a month but not every day)……2<br>About five times a week……..3<br>About three times a week……..4<br>Once in a week……5<br>Never………………6

Section 2: Structure of Lay Consultation Now, I want to ask you about how you discuss your health concerns or symptoms with members of your social networks, that is people who you are connected or related to, before seeking care or treatment.

**S/n**	**Questions**	**Response categories**
4	When did you last have an illness or any health concern that concerned you enough that you felt you needed some form of care or advice?	
5	What was the symptom or health concern?	
6	How did this symptom/health concern affect your daily functioning/activities?	
7	Did you discuss the symptom or health concern with anyone you are related or connected to, whether online or offline, or any online platform?	Yes ………………………1<br>No………………………..2
9	If yes, who or which platform did you first discuss the experience with?	a.
10	In all, how many people did you talk to?	
11	Did you discuss the illness/ health concern with anyone else or on any other platform? (asides the person you named in question 9) Please list them in the order in which you interacted with them	
		b
		c
		d
		e
		f
		g
		h
		i
12. Please, tell me more about each of the persons that you discussed with during this illness episode		

What is the person's initials/Nick Name	Which of the following best describes your relationship to (Name)?<br>Spouse (1)<br>Ex-spouse (2)<br>Friend (3)<br>Neighbor (4)<br>Romantic/sexual partner (5)<br>Co-worker or boss (6)<br>Parent (7)<br>Mother-in-law (8)<br>Father-in-law (9)<br>Child (10)<br>Brother or sister (11)<br>Other relative of yours (12)<br>Minister, priest or other clergy (13)<br>Medical Dr (14)<br>Community Health Worker (15)<br>Other (SPEC) (16)	Is (Name) male or female?<br>Male = 1<br>Female =2	How did you reach (Name)?<br>Face to face =1<br>Chat =2<br>Text message =3<br>E-mail = 4<br>Video call =5

How close do you live to (Name)?	What kind of medicine does (Name) Advocate?	Does (Name) have access to the internet?	Why did you discuss your symptom or health concern with (NAME)?	Which of the following was the main support you received from (NAME) for this illness episode?
Same household = 1<br>Same compound = 2<br>Same street = 4<br>Same LCDA = 5<br>Same state =6<br>Other (specify) = 7	Modern = 1<br>Traditional =2<br>Combination of Both =3<br>Others (specify) = 4	Yes =1<br>No =2<br>I don't know =3	To obtain information on the cause(s) of the symptom/health problem = 1<br>To ask for advice about formal health care provider = 2<br>To ask where to seek alternative care = 3<br>To learn about home remedies = 4<br>To report the illness =5<br>Because I am expected to tell (NAME) about my health condition?<br>Others (specify)------	Information on the causes/health problem (1)<br>Advice on where to seek care (2)<br>Information about formal health provider(4)<br>Information about alternative health provider (5)<br>Others (specify)

13	Did you seek care/treatment for the health concern?	Yes<br>No (if no, why?)
14	Which service/option did you use?	Medical doctor (including surgeon, gynaecologist, psychiatrist, ophthalmologist)………………………...1<br>Registered Nurse……………………………….2<br>Auxiliary nurse…………………………………3<br>Midwife……………………………….4<br>Dentist……………………………………5<br>Physiotherapist or chiropractor ………………6.<br>Traditional medicine practitioner ………………………7<br>Pharmacist, druggist……………………...8<br>Home remedy……………………………..9<br>Spiritualist…………………………………10<br>Self-medication……………………………….11<br>Other, please specify…………………………..12
**Section 3: Use of Digital Communication for lay consultation**<br>Now, I want to ask about how you use online platforms or forums to discuss your symptoms or health concern that you feel requires some form of care.		
15	Do you use online platform(s) to discuss your symptoms or health concern that you feel requires some form of care?	Yes……………………………………1<br>No……………………………………..2 (
16	If yes, which do you use?	(pick as many that applies to you)<br><br>Google………………………………………1<br>Bing…………………………………………2<br>Yahoo……………………………………..3<br>WebMD……………………………………4<br>Wikipedia……………………………………5<br>Facebook……………………………………6<br>You-tube……………………………………..7<br>Others, Please specify____________
17	In what ways do you share such health concerns/symptoms on online platforms?	Browsing……………………………1<br>Posting health related questions on health professional sites or social media platforms…2<br>Posting health related questions on platforms with persons personally known to you….3<br>Posting health related questions on sites/platforms with more general audience…3<br>Others (specify)……..
18	What do you post or share online?	Specific question about your health…………1<br>Comment……………………………………2<br>Story about your personal health experience...3<br>Other specify _____________________
**Section 4: Further Socio-demographic Characteristics of Respondents**<br>In this last part, I want to ask some further questions about yourself		

19	Employment	As you know, some people take up jobs for which they are paid in cash or kind. Others sell things, have a small business or work on the family farm or in the family business.<br>In the last seven days, have you done any of these things or any other work or have a business which you were absent from in the last seven days?	Yes...……………...1<br><br>No…………………….2
20	Marital status	What is your current marital status?	Married or living together……………1<br>Divorced / separated…………….2<br>Widowed………..…3<br>Never-married and Never lived together…….4
21	Medical Insurance	Are you under any kind of medical insurance?	Yes…………………..1<br>No……………………2
22	Migration status	Did you always live in this neighborhood?<br><br>(this question asks if the respondent was born here)	Yes…………..1<br>No…………….2

Table 2: Nature of illness or health concerns and perceived effects of illness or health concern on daily activities

**Nature of illness or health concern**		
**Infectious symptoms**		
Malaria	126	26.3
Malaria with headache/weakness/nausea/runny nose/ache	37	6.7
Typhoid	10	2.1
Measles-like rash	7	1.5
Malaria and typhoid	7	1.5
Malaria and ulcer	4	0.8
Typhoid and cough	1	0.2
Diarrhoea	2	0.4
Headache, cold and weakness	2	0.4
	196	40.8
**Headaches**		
Headache	55	11.5
Headache and body pain	5	1
Headache and weakness	3	0.8
Headache and fever	4	0.8
Headache and body ache	3	0.6
Headache and cold	1	0.2
Headache and catarrh	1	0.2
	72	15.0
**General pain and weakness**		
Body aches/body pain	34	6.3
Body weakness	25	5.2

Onuegbu C, *et al. BMJ Open* 2023; 13:e065152. doi: 10.1136/bmjopen-2022-065152

Shortage of blood	1	0.2
Body pain and weakness	2	0.4
Tiredness and temperature	1	0.2
Weakness and dizziness	2	0.4
	65	13.5
**Specific musculoskeletal pain**		
Leg pain/arm pain/shoulder/waist/thighs	29	4.4
Joint pain/knee pain	12	2.5
Chest pain	4	0.6
Back pain/ upper/lower	4	0.4
	49	10.2
**Gastrointestinal and abdominal issues**		
Piles	5	1
Stomach pain/lower abdominal pain/diarrhoea	17	3.5
Stomach ulcer	2	0.2
Stomach pain and headache	5	1
	29	6.0
**Others**		
Cough	5	1
Fever/high temperature	17	3.5
Accident/injuries/bruises	3	2.3
Catarrh	8	1.5
Menstrual cramps	3	0.8
Eye pain	4	0.8
Toothache	4	0.8
Pregnancy symptoms	4	0.8
Cough and catarrh	3	0.6
Anxiety/depression/sadness	3	0.6
Cataract/cloudy vision	3	0.6
High blood pressure	2	0.4
Miscarriage	2	0.4
Stress	2	0.4
Arthritis	2	0.4
Mouth ulcer	1	0.2
Dry throat	1	0.2
Vagina bleeding following menstruation	1	0.2
Fever and high bp	1	0.2
Fever, pile, and dizziness	1	0.2
Hernia surgery	1	0.2

Onuegbu C, *et al. BMJ Open* 2023; 13:e065152. doi: 10.1136/bmjopen-2022-065152

Stroke	1	0.2
Hypertension	1	0.2
Stiffness	1	0.2
Smelly burps	1	0.2
	69	14.4
Missing	1	0.2
**Perceived effect of illness/health concern on daily activities**	**N=480**	**%**
Some effects	266	55.4
No effects	214	44.6
**Kinds of effects**	**N=266**	**%**
Unable to go to work	82	30.8
Unable to perform normal household chores	59	22.2
Unable to work full time	30	11.3
Could not do any of my normal activities	39	14.7
Was on bed rest	24	9.1
Unable to perform religious activities	12	4.5
Could not exercise	8	3
Unable to sleep normally	4	1.5
Affected work and socialisation	4	1.5
Unable to socialise with friends	2	0.8
Hospitalised	1	0.4
Could not eat	1	0.4

Table 1. Association between relationship with network members and the reason for which they were consulted

		Reason for consultation		Chi-square	DF	P-value
		Consulting	Non-consulting			
Relationship	Family	39 (7.4%)	490 (92.6%)	50.781	1	0.000
	Non-family	45 (29.2%)	109 (70.8%)			

Table 2. Association between relationship with network members and support provided by network members

		Forms of support				X2	DF	P-value
		Information	Instrumental support	Emotional support	Appraisal			

Relationship	Family	198 (37.4%)	276 (52.2%)	31 (5.9%)	24 (4.5%)	16.499	3	0.001
	Non-family	79 (52.3%)	51 (33.8%)	10 (6.6%)	11 (7.3%)			
