# Supplementary material for: Use, characteristics and influence of lay consultation networks on treatment-seeking decisions in slums of Nigeria: a cross-sectional survey
Source: BMJ Open. 2023 May 16;13(5):e065152. doi: 10.1136/bmjopen-2022-065152 (PMC10193090; doi:10.1136/bmjopen-2022-065152)
Supplement: Author's manuscript [file bmjopen-2022-065152.draft_revisions.pdf]

BMJ Open

BMJ Open is committed to open peer review. As part of this commitment we make the peer review history of every article we publish publicly available.

When an article is published we post the peer reviewers' comments and the authors' responses online. We also post the versions of the paper that were used during peer review. These are the versions that the peer review comments apply to.

The versions of the paper that follow are the versions that were submitted during the peer review process. They are not the versions of record or the final published versions. They should not be cited or distributed as the published version of this manuscript.

BMJ Open is an open access journal and the full, final, typeset and author-corrected version of record of the manuscript is available on our site with no access controls, subscription charges or pay-per-view fees (http://bmjopen.bmj.com).

If you have any questions on BMJ Open's open peer review process please email info.bmjopen@bmj.com

BMJ Open

Use, characteristics, and influence of lay consultation networks on treatment-seeking decisions in slums of Nigeria: a cross-sectional survey

Journal:	*BMJ Open*
Manuscript ID	bmjopen-2022-065152
Article Type:	Original research
Date Submitted by the Author:	31-May-2022

Complete List of Authors:	Onuegbu, Chinwe ; University of Warwick, Health Sciences<br>Harlock, Jenny; University of Warwick<br>Griffiths, Frances; University of Warwick; University of the Witwatersrand

Keywords:	PUBLIC HEALTH, SOCIAL MEDICINE, PRIMARY CARE

SCHOLARONE™
Manuscripts

I, the Submitting Author has the right to grant and does grant on behalf of all authors of the Work (as defined in the below author licence), an exclusive licence and/or a non-exclusive licence for contributions from authors who are: i) UK Crown employees; ii) where BMJ has agreed a CC-BY licence shall apply, and/or iii) in accordance with the terms applicable for US Federal Government officers or employees acting as part of their official duties; on a worldwide, perpetual, irrevocable, royalty-free basis to BMJ Publishing Group Ltd ("BMJ") its licensees and where the relevant Journal is co-owned by BMJ to the co-owners of the Journal, to publish the Work in this journal and any other BMJ products and to exploit all rights, as set out in our licence.

The Submitting Author accepts and understands that any supply made under these terms is made by BMJ to the Submitting Author unless you are acting as an employee on behalf of your employer or a postgraduate student of an affiliated institution which is paying any applicable article publishing charge ("APC") for Open Access articles. Where the Submitting Author wishes to make the Work available on an Open Access basis (and intends to pay the relevant APC), the terms of reuse of such Open Access shall be governed by a Creative Commons licence – details of these licences and which Creative Commons licence will apply to this Work are set out in our licence referred to above.

Other than as permitted in any relevant BMJ Author's Self Archiving Policies, I confirm this Work has not been accepted for publication elsewhere, is not being considered for publication elsewhere and does not duplicate material already published. I confirm all authors consent to publication of this Work and authorise the granting of this licence.

For peer review only

TITLE: Use, characteristics, and influence of lay consultation networks on treatment-seeking decisions in slums of Nigeria: a cross-sectional survey

Corresponding author:

Chinwe Onuegbu

Division of Health Sciences, Warwick Medical School, University of Warwick, Coventry, CV4 7AL, UK

Chinwe.onuegbu@warwick.ac.uk

Co-authors:

Jenny Harlock[1] Jenny.Harlock@warwick.ac.uk

Frances Griffiths[1,2] F.E.Griffiths@warwick.ac.uk

[1]Division of Health Sciences, Warwick Medical School, University of Warwick, Coventry, CV4 7AL, UK

[2]Centre for Health Policy, University of the Witwatersrand, Johannesburg, South Africa

Word count: 2994

For peer review only

ABSTRACT

Objectives: To describe the use, characteristics, and influence of lay consultants on treatment-seeking decisions of adults in slums of Nigeria.

Design: Cross-section survey using a pre-piloted questionnaire

Settings: Two slum communities in Ibadan city, Nigeria

Participants: 480 adults within the working age group (18-64)

Results: Most respondents spoke to at least one lay consultant during their last illness/health concern (83.7%). All the lay consultants used were from personal networks; no respondent listed an online network or platform. About 9 in 10 persons reported conversations in which they discussed an illness/health concern without intending to seek any particular support. However, almost all the respondents obtained some form of support following the conversations (97%). Marital status ($P < 0.05$, OR 1.92, *95% CI* 1.10, 3.33) and perceiving that an illness or health concern had some effects on their daily activities ($P< 0.001$, OR 3.25, *95% CI* 1.94,5.46) had a significant independent association with speaking to at least one lay consultant. Age had a significant independent association with having lay consultation networks comprising non-family members only ($P< 0.01$, OR 0.95, *95% CI* 0.92, 0.99) or mixed networks (non-family and family members) ($P< 0.01$, OR 0.97, *95% CI* 0.95, 0.99), than family-only networks. Network characteristics influenced treatment decisions as having a network that comprised non-family member-only ($P < 0.01$, OR 0.23 *95% CI* 0.08, 0.67) and a combination of network members from the same household, neighbourhood and beyond ($P<0.05$, OR 2.04 95% CI 1.02, 4.09) were significantly associated with use of informal rather than formal healthcare.

Conclusions: Health programmes in urban slums should consider engaging community members so, when consulted within their networks, they are able to deliver reliable information about health and treatment-seeking.

Keywords: Slums, Informal settlements, lay consultants, lay referral system, social determinants of health

STRENGTHS AND LIMITATIONS

- This study examined the use of personal and online-based lay consultants, adding a distinctive approach to the literature, which is usually based on mapping either network.
- The study used name generators, which is a key social network technique, to generate information about networks.
- A robust and diverse sample was surveyed to ensure representativeness of different sociodemographics
- The survey examined the characteristics of networks and how these affect individual treatment decisions
- The study did not examine relationships between alters (individuals network members).

For peer review only - http://bmjopen.bmj.com/site/about/guidelines.xhtml

INTRODUCTION

The health of people living in slums is a global health priority.[1] About 60% of urban dwellers in sub-Saharan Africa live in slums.[1] Slums are low resource and overcrowded environments deficient in health-related infrastructures, including water, sanitation, and hygiene (WASH), adequate housing structure and secured tenure.[2] These conditions increase physical and mental health risks. Slum dwellers are exposed to negative neighbourhood effects that affect individual health beyond personal or household characteristics.[3,4] For example, formal and comprehensive healthcare facilities are scarce or inaccessible due to cost or distance, which contributes to the use of informal healthcare providers such as drug shops and alternative health practitioners.[5,6] Reliance on social support from informal sources such as personal network members are common in this contexts.[7]

Lay consultants are the personal network members (e.g. family and friends) or online sources/networks (e.g. websites, Facebook groups) that individuals interact with during an illness episode.[8,9] People speak to lay consultants for three main reasons: (i) to casually report a health concern without intending to seek support, (ii) to consult lay consultants for advice and information, and (iii) to seek instrumental forms of support, e.g. loans[10]. People are likely to engage different network members for specific reasons.[11] Some avoid discussing their health concerns with others for personal reasons, e.g. to maintain privacy.[12] Lay consultants provide various forms of support, including information or advice, appraisal of symptoms/health problems, instrumental support (e.g. cash) and emotional support (e.g. listening).[13] Provision of these resources has positive or negative consequences on personal treatment decisions.[14] For instance, lay consultants encourage or discourage people from seeking care from a formal healthcare provider.[15,16]

The characteristics of people's lay consultation networks influence the impact of the networks on treatment seeking decisions[14,17]. Network characteristics include network size (number of people consulted per illness episode), composition (characteristics of the group, e.g. proportion of kin) and culture (e.g. groups preference for formal healthcare practitioners). These characteristics are themselves influence by sociodemographic factors, including age, gender, marital status, residence and access to healthcare, all of which shape the use of personal networks. [13,18-21] For instance, women are more likely than men to speak to more people and have diverse networks per illness episode.[22,23] Thus, understanding network characteristics is important to understand their impact on behaviours.

For peer review only - http://bmjopen.bmj.com/site/about/guidelines.xhtml

Slum dwellers face socio-economic and environmental conditions that shape their personal and online networks. Being employed in informal labour with tight work conditions and frequent migration in slums contributes to small, closely-knit networks and limits networking with wider networks. [24,25] Living in clustered and intimately shared environments creates avenues for easy interaction,[26] but contributes to loss of individual privacy and friction among neighbours[27]. Digital gaps and low digital literacy is common among slum dwellers in low and middle-income countries,[28] which will likely affect access to online lay networks for advice.

There is empirical evidence suggesting slum dwellers interact with lay personal network members including family, friends and neighbours during illness and obtain a range of resources[5,7,29,30]. A recent systematic review demonstrated that lay consultation have positive and negative consequences on treatment seeking behaviours in slum settings of LMICs.[31] However, there are evidence gaps on network characteristics, how personal networks and online networks are combined, and how the network characteristics impact on personal health seeking decisions. Getting this evidence requires egocentric techniques, which are used in network studies to elicit information about a network from an ego (individual); this is lacking in slum studies.[7] This, this study responds to these evidence gaps and calls for more population-based studies from informal settlements of LMICs. [32,33]

Research objectives:

In the study conducted in slums in Nigeria, we focus on three objectives:

- To describe the use and characteristics of lay consultation networks (personal and online).
- To describe the factors that influence the size and composition lay consultation networks.
- To examine the association between characteristics of lay consultation networks and individual's decision to use formal or informal health services.

METHODS

Study design

NIHR slum health project

This survey builds on a multi-country project: the NIHR Global Research Unit on Improving Health in Slums[34] (the slum health project). As part of this project, household surveys were undertaken in seven sites across Nigeria, Kenya, Bangladesh, and Pakistan to investigate access

For peer review only - http://bmjopen.bmj.com/site/about/guidelines.xhtml

to and use of healthcare services. In Nigeria, the household survey was conducted in 2017 in one slum site in Lagos state and two in Oyo state.[35] Maps of the structures in the sites were generated and used to select samples for the household survey.[34] Findings from the Nigerian household survey can be accessed here.[35] We adopted a cross-sectional design and conducted the survey between November 2020 and January 2021.

Setting and participants

The survey was conducted in the two slum sites in Oyo State. The sites are anonymised to protect their identities. We recruited a sample of 480 adults between 18-64 years residing in the communities. We used the Cochrane sample size formula (p=0.5, CI=95%) with an attrition rate of 25%, to arrive at a sample size of 480. We recruited equal numbers of males and females across three age groups (18-29, 30-49 and 50-64) to ensure age/sex representation. To recruit the participants, we obtained a spatially referenced sampling frame listing all the structures/buildings in the two study communities, and randomly selected 480 structures. We then assigned a unique identifier and participant category (e.g. male 18-29, female 18-29) to each structure. Fieldworkers used Global Positioning System (GPS) to locate each structure to recruit the assigned participant category. In structures with more than one household, fieldworkers listed the households and randomly selected one to participate in the study. Similarly, when a household had more than one person who fit the participant category required, the persons were allotted unique identifiers and a lottery system was used to select one person. Where the assigned participant category was not found in the structure, the structure was recorded and automatically reassigned to another participant category.

Data collection

We collected data using a semi-structured questionnaire. The survey instrument contained questions adapted from previously validated questionnaires, including the Nigerian Demographic Health Survey (NDHS, 2018), the slum health project [34] and Pew research center (2013). We added questions developed from reviewing the literature[23]. The instrument comprised five parts with questions on:

1. Sociodemographic status of respondent;

2. A recent illness experience/health concern for which the respondent needed care or advice, their interactions with lay network members or lay online sources of health advice and types of healthcare used for this recent experience/concern.

For peer review only - http://bmjopen.bmj.com/site/about/guidelines.xhtml

3. Name-generating questions[36] – respondents asked to list all the lay network members or lay online sources of health advice for the recent experience/concern.

4. Socio-demographic characteristics, reasons for the interaction and support obtained from each name mentioned in 3.

5. General use of online platforms for advice seeking.

The study variables per each research objective is outlined in Supplementary table 1 in Supplementary file 1.

The instrument was translated into two local languages (Yoruba and Hausa) by linguists and then back-translated by different linguists to check consistency. Following this, it was reviewed at a community workshop in each field site, involving six community members selected through snowballing. We reworked the instrument and instrument guide based on their comments. We then pretested the instrument with a convenience sample of 20 participants residing just beyond our field sites. We analysed the responses using simple frequencies and percentages, and reviewed the questionnaire based on the results.

Trained fieldworkers administered the questionnaire using the Open-Data Kit (ODK), an open-source software tool that enables data collection, storage and analysis using mobile android devices. Respondents could choose to participate in the studies face-to-face or through mobile calls. The covid-19 public health guidelines provided by the Nigerian government were strictly followed for face-to-face data collection.

Data analysis

We calculated frequencies and simple percentages for all measures. We conducted bivariate analyses using chi-square to explore the association between network members individual characteristics with type of conversation they were consulted for and type of support they provided. We used bivariate logistic regression to analyse the association between personal characteristics of respondents and selected network characteristics (having at least one lay consultant and having non-family or mixed networks instead of family. We ran multivariable associations using a series of logistic regression models (i) individual characteristics and use of at least one lay consultant (ii) individual characteristics and having non-kin or mixed networks than family only networks (iii) network characteristics and use of formal health services while controlling for individual characteristics. The logistic model analyses were

For peer review only

performed at a 95% confidence interval limit, and a *P*-value less than 0.05 was deemed statistically significant. All statistical analysis was done using SPSS version 24.0.

Patient and public involvement

We involved residents and stakeholders from the communities in reviewing the instruments prior to the survey. Findings from this survey will be shared with residents and local lawmakers in the communities.

RESULTS

Sample characteristics

A total of 480 participants (240 per site) took part in the survey. Response rate was very high, as if eligible participants declined, they were replaced with others. Across the dataset, 98% of all questions were completed were completed. The sociodemographic characteristics of the study sample (see Table 1) was very similar to that of the population of the informal settlements as established in the slum health study.[28,37]

Just under half of the sample reported an illness/health concern that needed care or advice in the week preceding the survey. Infections were the common type of illness/health concern mentioned. Almost all the respondents used some form of care, and majority of them used informal healthcare practices/services. (see Table 1)

Table 1: Sociodemographic characteristics, last illness/health concern for which advice or care was needed and use of healthcare services

**Variable**	*N=480*	*(%)*
**Socio-demographics**		
Age categories		
18-29	176	36.7
30-49	166	34.6
50-64	138	28.8
Gender		
Male	223	46.5
Female	257	53.5
Marital status		
Married or living together	310	64.6
Not married	170	35.4
Employment status		
Currently employed	367	76.5
Not currently employed	113	24.5
Migration history		
Born in the neighbourhood	133	27.7
Moved from elsewhere	347	72.3
Daily mobile phone access		
Yes	432	90
No	48	10

For peer review only - http://bmjopen.bmj.com/site/about/guidelines.xhtml

For peer review only

Internet access		
At least once weekly	211	44
Never	269	56
Health insurance		
Yes	18	96.3
No	462	3.7
**Last illness experience**		
Last symptom experience		
Within the last week	225	(46.9)
Within the last month	136	(26.3)
Within the last three months	54	(11.3)
More than three months ago	75	(15.6)
Nature of illness or health concern	n=479[a]	
Infections (malaria, typhoid)	196	40.8
Headaches	72	15.0
General pain and weakness	65	13.5
Specific musculoskeletal pain	49	10.2
Gastrointestinal and abdominal issues	29	6.0
Others	69	14.4
Effect of symptom of health concern in daily functioning/activities	n=480	
Some effects	266	55.4
No effects	214	44.6
**Healthcare utilization**		
Sought care or treatment	n=480	
Yes	450	93.8
No	30	6.2
Type of health service used	n=450	
Formal medical care (e.g Medical doctors, nurses)	139	30.9
Non-formal care (e.g Patent Medicine Vendors, self medication)	311	69.1

[a]One non-response was recorded

Use and characteristics of lay consultation networks

Most respondents spoke to at least one lay consultant during their last illness/health concern. All the lay consultants used were from personal networks; no respondent listed an online network or platform. About 9 in 10 persons reported conversations in which they discussed an illness/health concern without intending to seek any particular support. However, almost all the respondents obtained some form of support following the conversations. Lay consultation networks mostly comprised one to two persons, family members only, and members of the same household or neighbourhood. Just over half of the sample had networks where no one had internet access. Only very few respondents said they had ever used online sources for seeking health advice. (see Table 2)

There was a significant association between relationship to network members and type of conversations initiated with them X^2 (1, N=683)=50.8, P<0.001. Respondents were

For peer review only - http://bmjopen.bmj.com/site/about/guidelines.xhtml

significantly more likely to engage in consulting conversations (in which they are asking for advice or information) with non-family members than family members (29.2 vs 7.4). Similarly, we found a significant association between relationship to network members and type of support obtained from them X^2 (3, N=683)=16.5, P<0.001. Non-family members were more likely than family members to provide information (52.3% vs 37.4%). A slightly higher proportion of non-family members than family members provided appraisal of symptoms (7.3% vs 4.5%) and emotional support (6.6% vs 5.9%). In contrast, Family members were more likely than non-family members to give instrumental support (52.2% vs 33.8%).

Table 2: Use and characteristics of the lay consultation network and general use of online sources for lay advice-seeking

**Variables**	**No**	**%**
**Use of lay consultants**		
Discussion with at least one person or platform	n=480	
Yes	400	83.7
No	80	16.3
**Characteristics of lay consultation/lay consultant**		
Relationship to all lay consultants[a]	n=683	
Kin	529	77.5
Non kin	154	22.5
Gender of all lay consultants[a]	N=683	
Male	294	43
Female	389	57
Means of communication with all lay consultants[a]	n=683	
Face to face	597	87
Mobile phone call	89	13
Reason for contacting lay consultant[a]	n=683	
Reporting	590	(86.4)
Consulting	84	(9.3)
Instrumental	9	(1.3)
Support obtained from lay consultants[a]	n=683	
Information	277	(40.7)
Appraisal	35	(6.0%)
Instrumental	327	(48.1)
Emotional	41	(5.1)
No support	3	(0.4)
**Structure of lay consultation networks**		
Lay consultation network size	n=480	
0	80	16.7
1	197	41
2	142	29.6
3	46	9.6
4	11	2.3
5	4	0.8
Relationship composition	n=400	
Family only	279	(69.8)
Non-family only	45	(11.3)
Mixed networks	76	(19.0)
Gender composition	n=400	
Women only	153	(38.3)

For peer review only - http://bmjopen.bmj.com/site/about/guidelines.xhtml

Men only	106	(26.5)
Mixed (women and men)	141	(35.3)
Geographic location of network members	n=400	
Household only	197	(49.3)
Neighbourhood only	34	(8.5)
Household or neighbourhood	46	(11.5)
Distant networks	47	(11.8)
Dispersed networks	76	(19.0)
Proportion of networks that used the internet	n=400	
0%	205	(51.2)
1-49%	15	(3.8)
50%	60	(15.0)
51-99%	20	(5.0)
100%	100	(25)
Medicine preference in the network	n=400	
All promote modern medicine only	170	42.5
All promote African Traditional Medicines (ATM) only	47	11.8
All promote combining modern medicine with ATM	72	18.0
Mixed medical culture	98	24.5
I don't know what they prefer	13	3.3
**General use of online sources for advice seeking**		
Ever used an online source for advice seeking	27	5.6
Never used an online source for advice seeking	453	94.4
If yes, which do you use?[b]	N= 27	
Google	19	70.4
Facebook	10	37.0
Youtube	4	14.8
In what ways do you share such health concerns/symptoms on online platforms?[b]		
Browsing	22	81.5
Posting health related questions on health professional sites or social media platforms	4	14.8
Posting health related questions on platforms of persons personally known to you	4	14.8
Posting health related questions on sites/platforms with more general audience	1	3.7
What do you post or share online? [b]		
Specific question about your health	4	14.8
Others- browse symptoms	23	85.2

[a] This is all the lay consultants contacted: 400 respondents contacted 683 lay consultants
[b] Percentage does not total 100 because it is a multichoice question

Association between individual characteristics and lay consultation network characteristics

Respondents who were currently employed, married, and thought that an illness or health concern had some effects on their daily activities were more likely to speak to at least one lay consultant during their last illness concern than those that were unemployed, unmarried or

For peer review only - http://bmjopen.bmj.com/site/about/guidelines.xhtml

thought the illness did not affect their everyday activities. Age, gender, marital status, and internet at least once a week were significantly associated with having non-family only networks than family-only networks. However, age and having internet at least once weekly were inversely associated with having a network composed exclusively of non-family members than family members. Age was the only individual characteristic associated with having a mixed network instead of a family-only network at the bivariate and multivariate levels. (see Table 3)

From the multivariable modelling we found marital status and perceiving that an illness or health concern had some effects on their daily activities has a significant independent association with speaking to at least one lay consultant (Table 4 Model 1). Only age has a significant independent association with non-family networks and mixed networks, respectively, than family-only networks (Table 4 Models 2 and 3).

Network characteristics and use of health services

Network variables influenced healthcare seeking, independent of personal characteristics (Table 4 Model 4). Respondents who interacted with non-family members only were less likely to use formal healthcare instead of informal healthcare than those who interacted with family-only networks and mixed networks. Those who interacted with network members in dispersed locations were more likely than those that spoke to household/neighbourhood and distant networks to use formal care instead of informal healthcare.

Table 3: Bivariate logistic regression results: relationship between personal characteristics and selected network characteristics

Personal characteristics	Having at least one lay consultant	Non-family only network[a]	Mixed networks[a]
Age	0.10 (0.98-1.01)	0.94 (0.91-0.97)***	0.97(0.95-0.99)**
Female	1.26 (0.78-2.04)	2.43 (1.25-4.71)**	0.63 (0.37-1.07)
Currently employed	0.52 (0.27-1.01)*	0.78 (0.36-1.69)	1.43 (0.82-2.50)
Currently married	0.62 (0.10-2.64)*	3.12 (1.64-5.95)***	0.99 (0.57-1.72)
Access to the internet at least once weekly	0.84 (0.52-1.36)	0.28 (0.143-0.55)***	0.73 (0.44-1.22)
Access to mobile phones daily	1.00 (0.45-2.23)	0.35 (0.80-1.50)	0.53 (0.20-1.39)
Access to health insurance	3.51 (0.46-26.73)	0.30 (0.87-1.05)	0.42 (0.13-1.32)

For peer review only - http://bmjopen.bmj.com/site/about/guidelines.xhtml

Perceived that an illness/health concern affected daily activities	3.12 (1.87-5.18)***	0.73 (0.37-1.47)	0.66 (0.39-1.12)

[a]Family-only network is the reference group
*P < 0.05, ** P<0.01, ***P<0.001

Table 4: Multivariable logistic regression results: relationship between personal characteristics and selected network characteristics; relationship between network characteristics and personal use of formal healthcare

	**At least one lay consultant**	**Non-family only networks[a]**	**Mixed networks[a]**	**Use of formal health care**
**Respondent's characteristics**	Model 1	Model 2	Model 3	Model 4
Age	0.99 (0.97-1.01)	0.95 (0.92-0.99)**	0.97 (0.95-0.99)**	1.01 (0.99-1.03)
Female	1.06 (0.63-1.79)	1.76 (0.85-3.61)	0.62 (0.35-1.09)	2.78(1.63-4.74)***
Currently employed	0.55 (0.28-1.10)	0.65 (0.28-1.54)	1.26 (0.70-2.27)	0.74 (0.43-1.27)
Currently married	1.92 (1.10-3.33)*	1.44 (0.64-3.25)	0.75 (0.40-1.42)	0.94 (054-1.65)
Access to the internet at least once weekly	0.74 (0.41-1.33)	0.73 (0.37-1.47)	1.07 (0.58-1.97)	2.37 (1.32-4.26)**
Access to mobile phones daily	1.27 (0.53-3.05)	0.49 (0.10-2.38)	0.46 (0.16-1.27)	2.92 (1.01-8.46)*
Access to health insurance	4.93 (0.62-38.98)	0.36 (0.09-1.45)	1.07 (0.58-1.97)	2.70 (0.85-8.60)
Perceived that an illness/health concern affected daily activities	3.25 (1.94-5.46)***	0.77 (0.38-1.56)	0.65 (0.38-1.13)	1.92 (1.16-3.18)**
**Network variables**				
Non-family only networks				0.23 (0.08-0.67)**
Mixed relationship networks				0.96 (0.50-1.86)
Women only networks				0.89 (0.46-1.71)
Mixed gender networks				0.87 (0.42-1.82)
Distant				1.95 (0.87-4.35)
Dispersed networks				2.04 (1.02-4.09)*
All network members promote formal health services				1.01(0.62-1.66)
At least one network member has internet				1.23 (0.74- 2.05)

[a]Family-only network is the reference group
*P < 0.05, ** P<0.01, ***P<0.001

For peer review only - http://bmjopen.bmj.com/site/about/guidelines.xhtml

DISCUSSION

This study demonstrated that respondents commonly interacted with lay consultants when they perceived an illness or health concern. The lay consultation networks used were small and dominated by personal network members characterised by face to face contacts. While we intended to describe both personal and online networks, we found that no respondent used online sources of lay advice and people rarely used the sources in general.

Small lay consultation networks dominated by family members have been found in non-slum settings in high-income settings.[17,21] Our finding that married respondents were likely to speak to a lay consultant supports previous evidence that spouses are important contacts in people's treatment pathway.[19,38] Respondents who thought a health concern affected their daily activities were likely to speak to a consultant. This is in line with published evidence that people are likely to initiate treatment-seeking decisions, part of which is speaking to lay consultants, for symptoms that affect their vocational and social activities.[39] Absence from work have particularly undesirable for slum dwellers who engage in daily informal labour for a daily wage[5] which makes it unsurprising that they engaged in lay consultation about illness concerns they perceived to affect their daily activities. As reported in other studies,[17,40] older persons were less likely to have non-family only networks or mixed networks. This relates to the socioemotional selectivity theory that an increase in age is associated with smaller social network sizes and network members that provide emotional support.[41]

Although our respondent's lay consultation networks were dominated by family members, they were more likely to specifically seek advice from non-family members than family members. This supports the functional specificity theory, [42] which suggests that people seek specific forms of support from their different networks. Our findings showed that non-family members were more likely than family members to provide information, while family members were more likely than non-family to provide instrumental support. Similarly, other studies suggest that friends and networks beyond one's immediate household are more likely to be sources of new information and ideas and family members are more likely to give hands-on support. [43,44] Our findings revealed respondents obtained appraisal of symptoms and emotional support from slightly more non-family members than family members. This is line with published evidence that show that peers are

important for evaluating and guiding others with similar symptoms and providing emotional support. [23,45]

Our findings that interaction with non-kin ties and extensive network members (combination of household/neighbourhood/distant network members) was significantly associated with using informal healthcare than formal healthcare is similar to what has been found in other non-slum contexts in a high-income country.[46] Weaker ties beyond the family and immediate environment can connect people to wider sources of information. [43] In our study case, lay consultants beyond the household may connect people to alternative forms of care not known within the family.

Our finding that no respondent used online sources contrasts with findings from research conducted in high-income countries where it is reported that internet sources were part of people's lay consultation network. [9,47,48] The contrast might be due to the digital and literacy gaps among slum dwellers in LMICs. [28]

Our survey was conducted in slum settings, which are hard-to-reach communities for researchers. [24] We used a robust sampling approach, which allowed us to capture data from a representative sample of adults from 18-64 years. In this study we did not ask respondents about the relationships between their network members.[36]

In summary, people living in urban slums interact with personal network members in their care-seeking pathways for illness, and the network members are important for how people decide on healthcare providers. Health programmes in urban slums should consider engaging community members so, when consulted within their networks, they are able to deliver reliable information about health and treatment-seeking.

Contributorship statement CO conducted the data analysis and wrote the entire article. JH and FG were involved in the design of the research, reviewed and revised the paper. All authors approved the final version of the paper.

Competing interest None

Funding

This research was funded by the National Institute for Health Research (NIHR) Global Health Research Unit on Improving Health in Slums using UK aid from the UK Government to support

For peer review only - http://bmjopen.bmj.com/site/about/guidelines.xhtml

global health research. The views expressed in this publication are those of the author(s) and not necessarily those of the NIHR or the UK Department of Health and Social Care. Grant reference number 16/136/87.

Data availability statement

Data are available upon reasonable request. The data are available from the study authors on request.

Ethics approval

All participants provided informed consent to participate before taking part in the study. Ethical approval was obtained from the Biomedical and Scientific Research Ethics Sub-Committee, University of Warwick, UK (BSREC 17/19-20 AM01) and the Research Ethics Committee of the Oyo State Ministry of Health, Nigeria (AD13/479/2035).

For peer review only

References

1. UN-Habitat. WORLD CITIES REPORT 2020: The value of sustainable urbanization. In: United Nations; 2020.
2. Un-Habitat. World cities report 2016: Urbanization and development–emerging futures. *United Nations Human Settlements Programme.* 2016.
3. Sunmola A, Alarape AI, Olusegun MA. The influence of house-ownership and the moderating effect of neighbourhood on life satisfaction among residents in Ibadan Metropolis, Nigeria. *IFE PsychologIA: An International Journal.* 2016;24(1):88-96.
4. Lilford RJ, Oyebode O, Satterthwaite D, et al. Improving the health and welfare of people who live in slums. *The lancet.* 2017;389(10068):559-570.
5. van der Heijden J, Gray N, Stringer B, et al. 'Working to stay healthy', health-seeking behaviour in Bangladesh's urban slums: a qualitative study. *BMC public health.* 2019;19(1):600.
6. Alaazi DA, Menon D, Stafinski T. Health, quality of life, and wellbeing of older slum dwellers in sub-Saharan Africa: A scoping review. *Global Public Health.* 2021;16(12):1870-1888.
7. Mpanje D, Gibbons P, McDermott R. Social capital in vulnerable urban settings: an analytical framework. *Journal of International Humanitarian Action.* 2018;3(1):4.
8. Reeder KM, Sims JL, Shetty SS. Lay consultations in symptom self-care: A concept analysis for theoretical expansion in research and practice. *Heart & Lung: The Journal of Cardiopulmonary and Acute Care.* 2015;44(6):548.
9. Loignon C, Gottin T, Rahem N, et al. Maternal Experience with Online Information on Parenting and Infant Care: Qualitative Findings from Quebec, Canada. *Journal of Child and Family Studies.* 2022:1-11.
10. Furstenberg AL, Davis LJ. Lay consulation of older people. *Social science & medicine.* 1984;18(10):827-837.
11. Perry BL, Pescosolido BA. Functional specificity in discussion networks: The influence of general and problem-specific networks on health outcomes. *Social Networks.* 2010;32(4):345-357.
12. Mwaka AD, Walter FM, Scott S, Harries J, Wabinga H, Moodley J. Symptom appraisal, help-seeking and perceived barriers to healthcare seeking in Uganda: an exploratory study among women with potential symptoms of breast and cervical cancer. *BMJ open.* 2021;11(2):e041365.
13. Reeder KM, Sims JL, Ercole PM, Shetty SS, Wallendorf M. Lay Consultations in Heart Failure Symptom Evaluation. *SOJ nursing & health care.* 2017;3(2).
14. Freidson E. *Profession of medicine: A study of the sociology of applied knowledge.* University of Chicago Press; 1988.
15. Smith D, Ruston A. 'If you feel that nobody wants you you'll withdraw into your own': Gypsies/Travellers, networks and healthcare utilisation. *Sociology of Health & Illness.* 2013;35(8):1196-1210.
16. Keboa MT, Hovey R, Nicolau B, Esfandiari S, Carnevale F, Macdonald ME. Oral healthcare experiences of humanitarian migrants in Montreal, Canada. *Canadian Journal of Public Health.* 2019;110(4):453-461.
17. Abbott KH, Stoller EP, Rose JH. The structure and function of frail male veterans' informal networks. *Journal of Aging and Health.* 2007;19(5):757-777.
18. Abbott KM, Bettger JP, Hanlon A, Hirschman KB. Factors associated with health discussion network size and composition among elderly recipients of long-term services and supports. *Health communication.* 2012;27(8):784-793.
19. Perry BL, Pescosolido BA. Social network activation: the role of health discussion partners in recovery from mental illness. *Social Science & Medicine.* 2015;125:116-128.

For peer review only - http://bmjopen.bmj.com/site/about/guidelines.xhtml

20. Pullen E, Oser C. Disadvantaged Status and Health Matters Networks among Low-Income African American Women. *Social Sciences.* 2017;6(3):108.
21. Queenan J, Gottlieb B, Feldman-Stewart D, Hall S, Irish J, Groome P. Symptom appraisal, help seeking, and lay consultancy for symptoms of head and neck cancer. *Psycho-oncology.* 2018;27(1):286-294.
22. Schoenberg NE, Amey CH, Stoller EP, Muldoon SB. Lay referral patterns involved in cardiac treatment decision making among middle-aged and older adults. *The Gerontologist.* 2003;43(4):493-502.
23. Stoller EP, Wisniewski AA. The structure of lay consultation networks: managing illness in community settings. *Journal of Aging and Health.* 2003;15(3):482-507.
24. van de Vijver S, Oti S, Oduor C, et al. Challenges of health programmes in slums. *The Lancet.* 2015;386(10008):2114-2116.
25. Gundewar A, Chin NP. Social capital, gender, and health: an ethnographic analysis of women in a Mumbai slum. *Global Health Promotion.* 2020:1757975920909114.
26. Vertigans S, Gibson N. Resilience and social cohesion through the lens of residents in a Kenyan informal settlement. *Community Development Journal.* 2020;55(4):624-644.
27. HU, A., LOO, E., WINCH, P. J. & SURKAN, P. J. 2012. Filipino women's tuberculosis care seeking experience in an urban poor setting: a socioecological perspective. *Health Care for Women International,* 33**,** 29-44.
28. Harris B, Ajisola M, Alam RM, et al. Mobile consulting as an option for delivering healthcare services in low-resource settings in low-and middle-income countries: A mixed-methods study. *Digital Health.* 2021;7:20552076211033425.
29. Das M, Angeli F, Krumeich AJ, van Schayck OC. Patterns of illness disclosure among Indian slum dwellers: a qualitative study. *BMC international health and human rights.* 2018;18(1):3.
30. Obembe T, Fonn S. Affording unavoidable emergency surgical care–The lived experiences and payment coping strategies of households in Ibadan metropolis, Southwestern Nigeria. *PloS one.* 2020;15(5):e0232882.
31. Onuegbu C, Larweh M, Harlock J, Griffiths F. Systematic review of lay consultation in symptoms and illness experiences in informal urban settlements of low-income and middle-income countries. *BMJ open.* 2021;11(12):e050766.
32. Vlahov D. Systematic surveys in informal settlements: challenges in moving toward health equity. *Journal of Urban Health.* 2019;96(6):795-796.
33. Satterthwaite D, Sverdlik A, Brown D. Revealing and responding to multiple health risks in informal settlements in sub-Saharan African cities. *Journal of urban health.* 2019;96(1):112-122.
34. Watson S. A protocol for a multi-site, spatially-referenced household survey in slum settings: methods for access, sampling frame construction, sampling, and field data collection. *BMC Medical Research Methodology.* 2019;19(1):109.
35. Fayehun O, Ajisola M, Uthman O, et al. A contextual exploration of healthcare service use in urban slums in Nigeria. *PLOS ONE.* 2022;17(2):e0264725.
36. Perry BL, Pescosolido BA, Borgatti SP. *Egocentric network analysis: Foundations, methods, and models.* Vol 44: Cambridge university press; 2018.
37. Collaborative IHiS. Inequity of healthcare access and use and catastrophic health spending in slum communities: a retrospective, cross-sectional survey in four countries. *BMJ Global Health.* 2021;6(11):e007265.
38. Cooper S, Gilbert L. The role of 'social support'in the experience of fibromyalgia–narratives from S outh A frica. *Health & social care in the community.* 2017;25(3):1021-1030.
39. Zola IK. Pathways to the doctor—from person to patient. *Social Science & Medicine (1967).* 1973;7(9):677-689.

For peer review only - http://bmjopen.bmj.com/site/about/guidelines.xhtml

40. Cook-Craig P, Ely G, Flaherty C, Dignan M, White CR. Seeking health advice from social networks in low-income urban neighborhoods. *American journal of health behavior.* 2012;36(6):723-735.
41. Lang FR, Staudinger UM, Carstensen LL. Perspectives on socioemotional selectivity in late life: How personality and social context do (and do not) make a difference. *The Journals of Gerontology Series B: Psychological Sciences and Social Sciences.* 1998;53(1):P21-P30.
42. Wellman B, Wortley S. Different strokes from different folks: Community ties and social support. *American journal of Sociology.* 1990;96(3):558-588.
43. Granovetter MS. The strength of weak ties. In: *Social networks.* Elsevier; 1977:347-367.
44. Roberts SG, Dunbar RI. The costs of family and friends: an 18-month longitudinal study of relationship maintenance and decay. *Evolution and Human Behavior.* 2011;32(3):186-197.
45. Hood S, Golembiewski E, Benbow K, Sow H, Sanders Thompson V. Who can I turn to? Emotional support availability in African American social networks. *Social Sciences.* 2017;6(3):104.
46. Schafer MH. Discussion networks, physician visits, and non-conventional medicine: probing the relational correlates of health care utilization. *Social Science & Medicine.* 2013;87:176-184.
47. Nettleton S, Hanlon G. 'Pathways to the Doctor'in the Information Age: the Role of ICTs in Contemporary Lay Referral Systems. In: *New technologies in health care.* Springer; 2006:57-70.
48. Sinha A, Porter T, Wilson A. The use of online health forums by patients with chronic cough: qualitative study. *Journal of medical Internet research.* 2018;20(1):e7975.

For peer review only

For peer review only - http://bmjopen.bmj.com/site/about/guidelines.xhtml

Supplementary Table 1 Study variables per research question

**Research questions**	**Variables (with definition for some)**	**Analysis method**	**Unit of analysis**
Participants sociodemographics and last illness experience	• Sociodemographic variables<br>• Last illness experience	Percentages and Frequency counts	Respondents
**<u>Research objective one:</u>** To describe use and characteristics of lay consultation networks?	**Personal characteristics of lay consultants:**<br>• Sociodemographic characteristics	Percentages and Frequency count	Lay consultants
	• Nature of ties to respondent	✓	✓
	• Means of communication	✓	✓
	• Main reason lay consultant was contacted/or could be contacted<br>○ Reporting<br>○ Consulting<br>○ Instrumental	Percentages, frequency counts and chi-square	✓
	• Type of support lay consultants provided/would potentially provide<br>○ Information/advice<br>○ Appraisal of symptoms<br>○ Instrumental<br>○ Emotional	Percentages, frequency counts and chi-square	✓
	**Network variables:**<br>• <u>Network size:</u> This is achieved by counting the number of network members listed by each	Percentages and Frequency counts	Networks

	respondent. There are three variables for network size<br>• Relationship composition<br>○ Family only networks<br>○ Non-family only networks<br>○ Mixed -relationship networks<br><br>• Gender composition<br>○ Women only networks<br>○ Men only networks<br>○ Mixed gender networks<br><br>• Geographical location of networks<br>○ Household only (all lay consultants are from the same household)<br>○ Neighbourhood only (all lay consultants are from the neighbourhood)<br>○ Household and neighbourhood<br>○ Distant networks (all lay consultants are located beyond the household or neighbourhood)<br>○ Dispersed networks (network combines lay combination from household, neighbourhood and distant locations<br>• Internet access in networks		

For peer review only - http://bmjopen.bmj.com/site/about/guidelines.xhtml

	○ 0% (no lay consultant in the network had access to the internet),<br>○ 1-49%,<br>○ 50%,<br>○ 51-99%,<br>○ 100% (all network members had access to the internet)<br>• Medical culture of networks<br>○ All lay consultants in the network promote modern medicine only<br>○ All lay consultants in the network promote African Traditional Medicines (ATM) only<br>○ All lay consultants in the network promote combining modern medicine with ATM<br>○ Combination of lay consultants that promote modern medicine, ATM and combining both.<br>○ I don't know what the lay consultants promote.		
	**General use of online sources of advice or support during illness**<br>• Types of platforms<br>• Style of communication<br>• Kind of posts.	Percentages and Frequency counts	Respondents

For peer review only - http://bmjopen.bmj.com/site/about/guidelines.xhtml

**<u>Research question two:</u>**<br><br>To describe the factors that influence the size and composition lay consultation networks.<br><br>.	a. Individual characteristics and having at least one lay consultant<br><u>Independent variables (Selected individual characteristics):</u><br><br>• Gender<br>○ Male = 0; Female = 1<br>• Age categories: in years<br>• Marital status:<br>○ Unmarried (comprising never married/never lived together, divorced/separated and widowed) = 0;<br>○ Married (married/living together) = 1<br>• Internet access:<br>○ No (never has internet) = 0;<br>○ Yes (comprising has internet everyday, about five times weekly, about three times weekly and once weekly) = 1.<br>• Daily mobile phone use:<br>○ No=0; Yes=1<br>• Current employment:<br>○ No =0, Yes = 1<br>• Perceived that illness/health concern affected daily activities:	a. Logistic regression (unadjusted odds ratio for bivariate analyses and adjusted odds ratio for multivariable analyses)<br><br>b. Multinomial logistic regression (unadjusted odds ratio for bivariate analyses and adjusted odds ratio for multivariable analyses).	

For peer review only - http://bmjopen.bmj.com/site/about/guidelines.xhtml

	○ No =0, Yes = 1<br><br><u>Dependent variables (Network variables)</u>:<br><br>• Having at least one lay consultant:<br>○ No = 0; Yes = 1<br><br>b. Individual characteristics and relationship in the network<br>• Independent variables same as (a) above<br>• <u>Dependent variables</u><br>○ Family-only networks 0; Non -family only networks = 1; mixed networks.		
**<u>Research objective three:</u>** To examine the association between characteristics of lay consultation networks and individual's decision to use formal or informal health services	<u>Independent variables (network variables):</u><br>○ Having at least one lay consultant: No =0 ; Yes =1<br>○ Relationship in the network<br>○ Family only network = 0; non-family only network =1; mixed relationship network= 2<br>○ Men only network =0; women only = 1; mixed gender network =2<br>○ Household/neighbourhood network = 0; distant network =1; dispersed network= 2<br>○ All network members promote use of formal healthcare: No =0; Yes =1	Adjusted odds ratio using bivariate logistic regression	

For peer review only - http://bmjopen.bmj.com/site/about/guidelines.xhtml

	Internet use in network: No (0% has internet) =0; At least one lay consultant has internet(comprising 1-49%, 50%, 51-99%, 100%) =1<br><br><u>Control variables (respondent's individual characteristics):</u><br><br>○ Individual characteristics listed in research question two above<br><br><u>Outcome variable (use of formal healthcare)</u><br><br>○ No=0<br>○ Yes = 1		

For peer review only

STROBE 2007 (v4) Statement—Checklist of items that should be included in reports of *cross-sectional studies*

Section/Topic	Item #	Recommendation	Reported on page #
**Title and abstract**	1	(*a*) Indicate the study's design with a commonly used term in the title or the abstract	1
		(*b*) Provide in the abstract an informative and balanced summary of what was done and what was found	2
**Introduction**			
Background/rationale	2	Explain the scientific background and rationale for the investigation being reported	3-4
Objectives	3	State specific objectives, including any prespecified hypotheses	4
**Methods**			
Study design	4	Present key elements of study design early in the paper	4-5
Setting	5	Describe the setting, locations, and relevant dates, including periods of recruitment, exposure, follow-up, and data collection	5
Participants	6	(*a*) Give the eligibility criteria, and the sources and methods of selection of participants	5
Variables	7	Clearly define all outcomes, exposures, predictors, potential confounders, and effect modifiers. Give diagnostic criteria, if applicable	Supplementary document 1
Data sources/ measurement	8*	For each variable of interest, give sources of data and details of methods of assessment (measurement). Describe comparability of assessment methods if there is more than one group	Supplementary document 1
Bias	9	Describe any efforts to address potential sources of bias	Not applicable
Study size	10	Explain how the study size was arrived at	5
Quantitative variables	11	Explain how quantitative variables were handled in the analyses. If applicable, describe which groupings were chosen and why	6-7
Statistical methods	12	(*a*) Describe all statistical methods, including those used to control for confounding	6-7
		(*b*) Describe any methods used to examine subgroups and interactions	Not applicable
		(*c*) Explain how missing data were addressed	Not applicable
		(*d*) If applicable, describe analytical methods taking account of sampling strategy	Not applicable
		(*e*) Describe any sensitivity analyses	Not applicable
**Results**			

For peer review only

Participants	13*	(a) Report numbers of individuals at each stage of study—eg numbers potentially eligible, examined for eligibility, confirmed eligible, included in the study, completing follow-up, and analysed	7
		(b) Give reasons for non-participation at each stage	
		(c) Consider use of a flow diagram	
Descriptive data	14*	(a) Give characteristics of study participants (eg demographic, clinical, social) and information on exposures and potential confounders	7
		(b) Indicate number of participants with missing data for each variable of interest	
Outcome data	15*	Report numbers of outcome events or summary measures	
Main results	16	(*a*) Give unadjusted estimates and, if applicable, confounder-adjusted estimates and their precision (eg, 95% confidence interval). Make clear which confounders were adjusted for and why they were included	7-13
		(*b*) Report category boundaries when continuous variables were categorized	Not applicable
		(*c*) If relevant, consider translating estimates of relative risk into absolute risk for a meaningful time period	Not applicable
Other analyses	17	Report other analyses done—eg analyses of subgroups and interactions, and sensitivity analyses	Not applicable
**Discussion**			
Key results	18	Summarise key results with reference to study objectives	14-15
Limitations	19	Discuss limitations of the study, taking into account sources of potential bias or imprecision. Discuss both direction and magnitude of any potential bias	15
Interpretation	20	Give a cautious overall interpretation of results considering objectives, limitations, multiplicity of analyses, results from similar studies, and other relevant evidence	15
Generalisability	21	Discuss the generalisability (external validity) of the study results	15
**Other information**			
Funding	22	Give the source of funding and the role of the funders for the present study and, if applicable, for the original study on which the present article is based	15

*Give information separately for cases and controls in case-control studies and, if applicable, for exposed and unexposed groups in cohort and cross-sectional studies.

Note: An Explanation and Elaboration article discusses each checklist item and gives methodological background and published examples of transparent reporting. The STROBE checklist is best used in conjunction with this article (freely available on the Web sites of PLoS Medicine at http://www.plosmedicine.org/, Annals of Internal Medicine at http://www.annals.org/, and Epidemiology at http://www.epidem.com/). Information on the STROBE Initiative is available at www.strobe-statement.org.

For peer review only

BMJ Open

Use, characteristics, and influence of lay consultation networks on treatment-seeking decisions in slums of Nigeria: a cross-sectional survey

Journal:	*BMJ Open*
Manuscript ID	bmjopen-2022-065152.R1
Article Type:	Original research
Date Submitted by the Author:	20-Mar-2023
Complete List of Authors:	Onuegbu, Chinwe ; University of Warwick, Health Sciences<br>Harlock, Jenny; University of Warwick<br>Griffiths, Frances; University of Warwick; University of the Witwatersrand
**Primary Subject Heading**:	Public health
Secondary Subject Heading:	Global health
Keywords:	PUBLIC HEALTH, SOCIAL MEDICINE, PRIMARY CARE

SCHOLARONE™ Manuscripts

I, the Submitting Author has the right to grant and does grant on behalf of all authors of the Work (as defined in the below author licence), an exclusive licence and/or a non-exclusive licence for contributions from authors who are: i) UK Crown employees; ii) where BMJ has agreed a CC-BY licence shall apply, and/or iii) in accordance with the terms applicable for US Federal Government officers or employees acting as part of their official duties; on a worldwide, perpetual, irrevocable, royalty-free basis to BMJ Publishing Group Ltd ("BMJ") its licensees and where the relevant Journal is co-owned by BMJ to the co-owners of the Journal, to publish the Work in this journal and any other BMJ products and to exploit all rights, as set out in our licence.

The Submitting Author accepts and understands that any supply made under these terms is made by BMJ to the Submitting Author unless you are acting as an employee on behalf of your employer or a postgraduate student of an affiliated institution which is paying any applicable article publishing charge ("APC") for Open Access articles. Where the Submitting Author wishes to make the Work available on an Open Access basis (and intends to pay the relevant APC), the terms of reuse of such Open Access shall be governed by a Creative Commons licence – details of these licences and which Creative Commons licence will apply to this Work are set out in our licence referred to above.

Other than as permitted in any relevant BMJ Author's Self Archiving Policies, I confirm this Work has not been accepted for publication elsewhere, is not being considered for publication elsewhere and does not duplicate material already published. I confirm all authors consent to publication of this Work and authorise the granting of this licence.

For peer review only

TITLE: Use, characteristics, and influence of lay consultation networks on treatment-seeking decisions in slums of Nigeria: a cross-sectional survey

Corresponding author:

Chinwe Onuegbu

Division of Health Sciences, Warwick Medical School, University of Warwick, Coventry, CV4 7AL, UK

Chinwe.onuegbu@warwick.ac.uk

Co-authors:

Jenny Harlock[1] Jenny.Harlock@warwick.ac.uk

Frances Griffiths[1,2] F.E.Griffiths@warwick.ac.uk

[1]Division of Health Sciences, Warwick Medical School, University of Warwick, Coventry, CV4 7AL, UK

[2]Centre for Health Policy, University of the Witwatersrand, Johannesburg, South Africa

Word count: 3741

For peer review only

For peer review only - http://bmjopen.bmj.com/site/about/guidelines.xhtml

ABSTRACT

Objectives: To describe the use, characteristics, and influence of lay consultants on treatment-seeking decisions of adults in slums of Nigeria.

Design: Cross-section survey using a pre-piloted questionnaire

Settings: Two slum communities in Ibadan city, Nigeria

Participants: 480 adults within the working age group (18-64)

Results: Most respondents (400/480, 83.7%) spoke to at least one lay consultant during their last illness/health concern. In total, 683 lay consultants were contacted; all from personal networks such as family and friends. No respondent listed online network members or platforms. About 9 in 10 persons spoke to a lay consultant about an illness/health concern without intending to seek any particular support. However, almost all (680/683, 97%) lay consultants that were contacted provided some form of support. Marital status (OR = 1.92, *95%* CI: 1.10- 3.33) and perceiving that an illness or health concern had some effects on their daily activities (OR = 3.25, *95%* CI: 1.94 - 5.46) had a significant independent association with speaking to at least one lay consultant. Age had a significant independent association with having lay consultation networks comprising non-family members only (OR = 0.95, *95%* CI: 0.92 - 0.99) or mixed networks (non-family and family members) (OR = 0.97, *95%* CI: 0.95 - 0.99), rather than family-only networks. Network characteristics influenced individual treatment decisions as participants who contacted networks comprising non-family members only (OR=0.23, *95%* CI*:* 0.08 - 0.67) and dispersed networks (combination of household, neighbourhood and distant network members) (OR = 2.04, 95% CI: 1.02 - 4.09) were significantly more likely to use informal than formal healthcare, while controlling for individual characteristics.

Conclusions: Health programmes in urban slums should consider engaging community members so, when consulted within their networks, they are able to deliver reliable information about health and treatment-seeking.

Keywords: Slums, Informal settlements, lay consultants, lay referral system, social determinants of health

STRENGTHS AND LIMITATIONS

- To the best of our knowledge, this is the first survey to extensively describe the use, characteristics, and influence of lay consultation networks on management of illnesses and health concerns in slum settings.
- The study was conducted among adults recruited through a robust sampling strategy in slum communities which are hard-to-reach communities for researchers
- This study uses an established social network research technique for identifying egocentric networks. This is a novel application and makes a unique contribution to the literature on slum health in Low and Middle-income Countries
- The data supplied by respondents about their network members were not independently verified by interviewing the network members.
- There may have been recall bias as participants were asked to recall information about their illness concerns and use of networks, however the short recall period minimises this risk.

For peer review only - http://bmjopen.bmj.com/site/about/guidelines.xhtml

INTRODUCTION

The health of people living in slums is a global health priority.(1) About 60% of urban dwellers in sub-Saharan Africa live in slums.(1) Slums are low resource and overcrowded environments deficient in health-related infrastructures, including water, sanitation, and hygiene (WASH), adequate housing structure and secured tenure.(2) These conditions increase physical and mental health risks. Slum dwellers are exposed to negative neighbourhood effects that affect individual health beyond personal or household characteristics.(3,4) For example, formal and comprehensive healthcare facilities are scarce or inaccessible due to cost or distance, which contributes to the use of healthcare providers such as drug shops and alternative health practitioners.(5,6) Reliance on social support from informal sources such as personal network members are common in these contexts.(7)

Lay consultants are the personal network members (e.g. family and friends) or online sources/networks (e.g. websites, Facebook groups) that individuals interact with during an illness episode.(8,9) People speak to lay consultants for three main reasons: (i) to casually report a health concern without intending to seek support, (ii) to consult lay consultants for advice and information, and (iii) to seek instrumental forms of support, e.g. loans.(10) People are likely to engage different network members for specific reasons.(11) Some avoid discussing their health concerns with others for personal reasons, e.g. to maintain privacy.(12) Lay consultants provide various forms of support, including information or advice, appraisal of symptoms/health problems, instrumental support (e.g. cash) and emotional support (e.g. listening).(13) Provision of these resources has positive or negative consequences on personal treatment decisions.(14) For instance, lay consultants encourage or discourage people from seeking care from a formal healthcare provider.(15,16)

The characteristics of people's lay consultation networks influence the impact of the networks on treatment seeking decisions.(14,17) Network characteristics include network size (number of people consulted per illness episode), composition (characteristics of the group, e.g. proportion of kin) and culture (e.g. groups preference for formal healthcare practitioners). These characteristics are themselves influenced by sociodemographic factors, including age, gender, marital status, residence, and access to healthcare, all of which shape the use of personal networks.(13,18-21) For instance, women are more likely than men to speak to more people and have diverse networks per illness episode.(22,23) Thus, understanding network characteristics is important to understand their impact on behaviours.

For peer review only - http://bmjopen.bmj.com/site/about/guidelines.xhtml

Slum dwellers face socio-economic and environmental conditions that shape their personal and online networks. Being employed in informal labour with tight work conditions and frequent migration in slums contributes to small, closely-knit networks and limits networking with wider networks.(24,25) Living in clustered and intimately shared environments creates avenues for easy interaction,(26) but contributes to loss of individual privacy and friction among neighbours.(27) Digital gaps and low digital literacy is common among slum dwellers in low and middle-income countries,(28) which will likely affect access to online lay networks for advice.

There is empirical evidence suggesting slum dwellers interact with lay personal network members including family, friends and neighbours during illness and obtain a range of resources.(5,7,29,30) A recent systematic review demonstrated that lay consultation have positive and negative consequences on treatment seeking behaviours in slum settings of LMICs .(31) However, there are evidence gaps on network characteristics, how personal networks and online networks are combined, and how the network characteristics impact on personal health seeking decisions. Getting this evidence requires egocentric techniques, which are used in network studies to elicit information about a network from an ego (individual); this is lacking in slum studies.(7) This, this study responds to these evidence gaps and calls for more population-based studies from informal settlements of LMICs.(32, 33)

Research objectives:

In the study conducted in slums in Nigeria, we focus on three objectives:

- To describe the use and characteristics of lay consultation networks (personal and online).
- To describe the factors associated with the size and composition lay consultation networks.
- To examine the association between characteristics of lay consultation networks and individual's decision to use formal or informal health services.

For peer review only - http://bmjopen.bmj.com/site/about/guidelines.xhtml

METHODS

Study design

<u>NIHR slum health project: Surveys undertaken prior to this study</u>

This survey builds on a multi-country project: the NIHR Global Research Unit on Improving Health in Slums(the slum health project).(34) The slum health project involved household surveys across seven slums in Nigeria, Kenya, Bangladesh, and Pakistan. The primary aim to investigate access to and use of healthcare services. The household survey in Nigeria was conducted in 2017, in one slum in Lagos state and two slums in Oyo state.(35) Maps of the structures in the sites were generated and used to select samples for the household survey.(34) Findings from the Nigerian household survey can be accessed here.(35)

The survey reported in this article builds upon the NIHR slum health survey in the two slum areas in Oyo state, Nigeria.

We adopted a cross-sectional design and conducted the survey between November 2020 and January 2021. We used an egocentric survey design to elicit data about networks. An egocentric survey involves using a name generator to ask a participant (ego) about their network members (alters) and the relationship between them and their network members.(36) This differs from a sociocentric study where information is obtained from egos and alters.(36) Both designs are common in network studies and are selected based on the research question. For this study on people's perception of their use of lay consultation networks, an egocentric design was appropriate.

The survey was followed by qualitative research.(37)

Setting and participants

The survey was conducted in the two slum sites located in Ibadan, Oyo State. The sites are anonymised to protect their identities. The slums were selected as they met the definition of urban slums, and were accessible based on negotiations by the prior NIHR slum health survey.(34) Ibadan is one of the largest indigenous cities in Africa.(38) The city has a an annual population growth rate of around 2.39% and a current population of about 3 million.(38,39) Slums in Ibadan are characterised by poor housing quality, tenure insecurity, insecurity of lives and property, and the absence of basic infrastructures, including water supply and proper housing structures.(40,41) One of the slums suveryed was a migrant community of around 5800 persons, most of whom are young and involved in trading. The second slum was an indigenous slums inabiting around 5,500 persons. Both slums have a high presence of

For peer review only - http://bmjopen.bmj.com/site/about/guidelines.xhtml

informal healthcare providers including Patent Medicine Vendors and traditional/spiritual healers.(35)

We recruited a sample of 480 adults between 18-64 years residing in the communities. The criteria for inclusion included: being aged 18 to 64, and residing in the community. We used the Cochrane sample size formula (p=0.5, CI=95%) with an attrition rate of 25%, to arrive at a sample size of 480. We recruited equal numbers of males and females across three age groups (18-29, 30-49 and 50-64) to ensure age/sex representation. To recruit the participants, we obtained a spatially referenced sampling frame listing all the structures/buildings in the two study communities, and randomly selected 480 structures. We then assigned a unique identifier and participant category (e.g. male 18-29, female 18-29) to each structure. Fieldworkers used Global Positioning System (GPS) to locate each structure to recruit the assigned participant category. In structures with more than one household, fieldworkers listed the households and randomly selected one to participate in the study. Similarly, when a household had more than one person who fit the participant category required, the persons were allotted unique identifiers and a lottery system was used to select one person. Where the assigned participant category was not found in the structure, the structure was recorded and automatically reassigned to another participant category. During the recruitment, three people decided not to take part, and they were replaced with three other participants.

Data collection

We collected data using a semi-structured questionnaire (see supplementary file, table 1). The survey instrument contained questions adapted from previously validated questionnaires, including the Nigeria Demographic Health Survey (NDHS, 2018),(42) the slum health project,(34) and Pew research center (2013).(43) We added questions developed from reviewing the literature.(23) The instrument comprised five parts with questions on:

1. Sociodemographic status of respondent;

2. A recent illness experience/health concern for which the respondent needed care or advice (open-ended question), their interactions with lay network members or lay online sources of health advice and types of healthcare used for this recent experience/concern.

3. Name-generators(36) – respondents asked to list all the lay network members or lay online sources of health advice for the recent experience/concern.

For peer review only - http://bmjopen.bmj.com/site/about/guidelines.xhtml

4. Socio-demographic characteristics, reasons for the interaction and support obtained from each name mentioned in 3.

5. General use of online platforms for advice seeking.

The study variables per each research objective is outlined in Supplementary table 1 in Supplementary file 1.

The instrument was translated into two local languages (Yoruba and Hausa) by linguists and then back-translated by different linguists to check consistency. Following this, it was reviewed at a community workshop in each field site, involving six community members selected through snowballing. We reworked the instrument and instrument guide based on their comments. We then pretested the instrument with a convenience sample of 20 participants residing just beyond our field sites. We analysed the responses using simple frequencies and percentages, and reviewed the questionnaire based on the results.

Trained fieldworkers administered the questionnaire using the Open-Data Kit (ODK), an open-source software tool that enables data collection, storage and analysis using mobile android devices. Respondents could choose to participate in the studies face-to-face or through mobile calls. The covid-19 public health guidelines provided by the Nigerian government were strictly followed for face-to-face data collection.

Data analysis

The questions on the recency of an illness or health concern experienced by respondents, nature of illness or health concern, and how the illness or health concern affected people's daily activities/functioning were open ended questions. Responses were first content analysed and then put into categories.

We calculated frequencies and simple percentages for all measures. We conducted bivariate analyses using chi-square to explore the association between network members individual characteristics with type of conversation they were consulted for and type of support they provided.

We ran multivariable associations using a series of logistic regression models

(i) individual characteristics and use of at least one lay consultant

(ii) individual characteristics and having non-kin or mixed networks than family only networks

For peer review only - http://bmjopen.bmj.com/site/about/guidelines.xhtml

(iii) network characteristics and use of formal health services while controlling for individual characteristics.

The logistic model analyses were performed at a 95% confidence interval limit, and a *P*-value less than 0.05 was deemed statistically significant. All statistical analysis was done using SPSS version 24.0.

Patient and public involvement

We involved residents and stakeholders from the communities in reviewing the instruments prior to the survey. Findings from this survey will be shared with residents and local lawmakers in the communities.

RESULTS

Sample characteristics

A total of 480 participants (240 per site) took part in the survey. Response rate was very high at 99.4% (attrition rate was 0.6%, 3/480). Across the dataset, 98% of all questions were completed were completed. The sociodemographic characteristics of the study sample (see Table 1) was very similar to that of the population of the informal settlements as established in the slum health study.(28,44)

Just under half of the sample reported they experienced an illness/health concern in the week preceding the survey, and that they felt that they need needed care or advice for it. Of these, 196 (40.8%) reported illness of health concern was an infection(144 specified malaria). Other illness/health concerns were headaches, general pain and weakness, specific musculoskeletal pain, Gastrointestinal and abdominal issues, and 'others' (Please see supplementary file table 2for list of illnesses reported). Almost all the respondents used some form of care, and majority of them used informal healthcare practices/services. (see Table 1)

Table 1: Sociodemographic characteristics, last illness/health concern for which advice or care was needed and use of healthcare services

**Variable**	N=480	(%)
**Socio-demographics**		
Age categories	Mean 37.8, S.D 14	
18-29	176	36.7
30-49	166	34.6
50-64	138	28.8
Gender		
Male	223	46.5

For peer review only - http://bmjopen.bmj.com/site/about/guidelines.xhtml

Female	257	53.5
Marital status		
Married or living together	310	64.6
Not married	170	35.4
Employment status		
Currently employed	367	76.5
Not currently employed	113	24.5
Migration history		
Born in the neighbourhood	133	27.7
Moved from elsewhere	347	72.3
Daily mobile phone access		
Yes	432	90
No	48	10
Internet access		
At least once weekly	211	44
Never	269	56
Health insurance		
Yes	18	96.3
No	462	3.7
**Last illness/health concern experience**		
Last illness/health concern experience	n=480	
Within the last week	225	(46.9)
Within the last month	136	(26.3)
Within the last three months	54	(11.3)
More than three months ago	75	(15.6)
Nature of illness or health concern	n=479[a]	
Infections (malaria, typhoid)	196	40.8
Headaches	72	15.0
General pain and weakness	65	13.5
Specific musculoskeletal pain	49	10.2
Gastrointestinal and abdominal issues	29	6.0
Others	69	14.4
Effect of symptom of health concern in daily functioning/activities	n=480	
Some effects	266	55.4
No effects	214	44.6
**Healthcare utilization**		
Sought care or treatment	n=480	
Yes	450	93.8
No	30	6.2
**Type of health service used**	n=450	
**Formal healthcare services (**Medical Drs/Registered Nurse/Physiotherapists**)**	107	23.8
**Non-formal care:** Patent medicine vendors	209	46.4
Self-medication	43	9.6
Pharmacist	32	7.1
Auxiliary nurse	27	6
Home remedy	17	3.8
Traditional medicine practitioner	13	2.9
Spiritualist	2	0.4

[a]One non-response was recorded

For peer review only - http://bmjopen.bmj.com/site/about/guidelines.xhtml

Use and characteristics of lay consultation networks

Most respondents spoke to at least one lay consultant during their last illness/health concern. All the lay consultants used were from personal networks; no respondent listed an online network or platform. About 9 in 10 persons reported conversations in which they discussed an illness/health concern without intending to seek any particular support. However, almost all the respondents obtained some form of support following the conversations. Lay consultation networks mostly comprised one to two persons, family members only, and members of the same household or neighbourhood. The lay consultants included spouses/partners (204/683, 29.9%), friends/neighbours 135/683, 19.7), children (131/683. 19.2%), parent (124/683. 18.2%), other relatives (76/683, 11.2%), co-workers (10/683, 1.5%) and clergy (3/683. 0.4%).

Just over half of the sample had networks where no one had internet access. Only very few respondents (27/480, 5.6%) said they had ever used online sources for seeking health advice. (see Table 2).There was a significant association between relationship to network members and type of conversations held with them X^2 (1, N=683)=50.8, P<0.001). Respondents were significantly more likely to engage in consulting conversations (in which they are asking for advice or information) with non-family members than family members (29.2 vs 7.4). Similarly, we found a significant association between relationship to network members and type of support obtained from them X^2 (3, N=683)=16.5, P<0.001). Non-family members were more likely than family members to provide information (52.3% vs 37.4%). A slightly higher proportion of non-family members than family members provided appraisal of symptoms (7.3% vs 4.5%) and emotional support (6.6% vs 5.9%). In contrast, Family members were more likely than non-family members to give instrumental support (52.2% vs 33.8%). These results are presented in Supplementary file table 3 and 4.

Table 2: Use and characteristics of the lay consultation network and general use of online sources for lay advice-seeking

Variables	No	%
**Use of lay consultants**		
Discussion with at least one person (online or offline) or platform	n=480	
Yes	400	83.7
No	80	16.3
**Characteristics of lay consultation/lay consultant**		
Relationship to all lay consultants[a]	n=683	
Kin	529	77.5
Non kin	154	22.5
Kind of relationship to lay consultant	n=683	

For peer review only - http://bmjopen.bmj.com/site/about/guidelines.xhtml

Spouse/ partner	204	29.9
Friend/neighbour	135	19.7
Child	131	19.2
Parent	124	18.2
Other relative	76	11.2
Co-worker	10	1.5
Clergy	3	0.4
Gender of all lay consultants[a]	n=683	
Male	294	43
Female	389	57
Means of communication with all lay consultants[a]	n=683	
Face to face	597	87
Mobile phone call	89	13
Reason for contacting lay consultant[a]	n=683	
Reporting	590	(86.4)
Consulting	84	(9.3)
Instrumental	9	(1.3)
Support obtained from lay consultants[a]	n=683	
Information	277	(40.7)
Appraisal	35	(6.0%)
Instrumental	327	(48.1)
Emotional	41	(5.1)
No support	3	(0.4)
**Structure of lay consultation networks**		
Lay consultation network size	n=480	
0	80	16.7
1	197	41
2	142	29.6
3	46	9.6
4	11	2.3
5	4	0.8
Relationship composition	n=400	
Family only	279	(69.8)
Non-family only	45	(11.3)
Mixed networks	76	(19.0)
Gender composition	n=400	
Women only	153	(38.3)
Men only	106	(26.5)
Mixed (women and men)	141	(35.3)
Geographic location of network members	n=400	
Household only	197	(49.3)
Neighbourhood only	34	(8.5)
Household or neighbourhood	46	(11.5)
Distant networks	47	(11.8)
Dispersed networks (all are located beyond the household and neighbourhood)	76	(19.0)
Proportion of networks that used the internet	n=400	
At least one network member had internet access	195	48.8
None of the network members had network access	205	(51.2)
Medicine preference in the network	n=400	
All promote modern medicine only: Yes<br>No	170<br>230	42.5<br>57.5
**General use of online sources for advice seeking**		
Ever used an online source for advice seeking	27	5.6
Never used an online source for advice seeking	453	94.4

For peer review only - http://bmjopen.bmj.com/site/about/guidelines.xhtml

If yes, which do you use?[b]	N= 27	
Google	19	70.4
Facebook	10	37.0
Youtube	4	14.8
In what ways do you share such health concerns/symptoms on online platforms?[b]		
Browsing	22	81.5
Posting health related questions on health professional sites or social media platforms	4	14.8
Posting health related questions on platforms of persons personally known to you	4	14.8
Posting health related questions on sites/platforms with more general audience	1	3.7
What do you post or share online? [b]		
Specific question about your health	4	14.8
Others- browse symptoms	23	85.2

[a] This is all the lay consultants contacted: 400 respondents contacted 683 lay consultants
[b] Percentage does not total 100 because it is a multichoice question

Factors associated with the use, size and composition of participants lay consultation networks

Being married (OR= 1.92; *95%* CI: 1.10-3.33) and perceiving that an illness or health concern had some effects on their daily activities (OR= 3.25; *95%* CI: 1.94-5.46) has a significant independent association with speaking to at least one lay consultant (see Table 4 Model 1). Age had a significant independent association with non-family networks (OR=0.95; *95%* CI: 0.92-0.99) and mixed networks (OR=0.97; *95%* CI: 0.95-0.99) than family-only networks (see Table 3 Models 2 and 3, respectively).

Table 3: Multivariable logistic regression results: relationship between personal characteristics and selected network characteristics; relationship between network characteristics and personal use of formal healthcare

	**At least one lay consultant**	**Non-family only networks[a]**	**Mixed networks[a]**
**Respondent's characteristics**	**Model 1 OR (95% CI)**	**Model 2 OR (95% CI)**	**Model 3 OR (95% CI)**
Age	0.99 (0.97-1.01)	0.95 (0.92-0.99)**	0.97 (0.95-0.99)**
Female	1.06 (0.63-1.79)	1.76 (0.85-3.61)	0.62 (0.35-1.09)
Currently employed	0.55 (0.28-1.10)	0.65 (0.28-1.54)	1.26 (0.70-2.27)
Currently married	1.92 (1.10-3.33)*	1.44 (0.64-3.25)	0.75 (0.40-1.42)
Access to the internet at least once weekly	0.74 (0.41-1.33)	0.73 (0.37-1.47)	1.07 (0.58-1.97)
Access to mobile phones daily	1.27 (0.53-3.05)	0.49 (0.10-2.38)	0.46 (0.16-1.27)
Access to health insurance	4.93 (0.62-38.98)	0.36 (0.09-1.45)	1.07 (0.58-1.97)

For peer review only - http://bmjopen.bmj.com/site/about/guidelines.xhtml

Perceived that an illness/health concern affected daily activities	3.25 (1.94 -5.46)***	0.77 (0.38-1.56)	0.65 (0.38-1.13)

[a]Family-only network is the reference group
*P < 0.05, ** P<0.01, ***P<0.001

Network characteristics and use of health services

Table 4 shows that network variables influenced healthcare seeking, independent of personal characteristics. Respondents who interacted with non-family members only (OR= 0.23, *95%* CI: 0.08-0.67) were less likely to use formal healthcare instead of informal healthcare than those who interacted with family-only networks and mixed networks. Those who interacted with network members in dispersed locations (OR= 2.04, *95%* CI: 1.02-4.09) were more likely than those that spoke to household/neighbourhood and distant networks to use formal care instead of informal healthcare.

Table 4: Multivariate analyses exploring the association between network variables and use of formal healthcare while controlling for age, gender, employment, marital status, internet access, health insurance and perceiving that an illness affects daily activities

**Network variables**	**Use of formal care OR (95% CI)**
Non-family only networks	0.23 (0.08-0.67)**
Mixed relationship networks	0.96 (0.50-1.86)
Women only networks	0.89 (0.46-1.71)
Mixed gender networks	0.87 (0.42-1.82)
Distant networks	1.95 (0.87-4.35)
Dispersed networks	2.04 (1.02-4.09)*
All network members promote formal health services	1.01(0.62-1.66)
At least one network member has internet	1.23 (0.74- 2.05)

[a]Family-only network is the reference group
*P < 0.05, ** P<0.01, ***P<0.001

DISCUSSION

This study demonstrated that respondents commonly interacted with lay consultants when they perceived an illness or health concern. The lay consultation networks used were small and dominated by personal network members characterised by face-to-face contacts. While we intended to describe both personal and online networks, we found that no respondent used

For peer review only - http://bmjopen.bmj.com/site/about/guidelines.xhtml

online sources of lay advice and people rarely used them when managing illnesses or health concerns.

The finding that most people with illness symptoms or health concerns spoke to at least one network member before seeking formal or informal care is unsurprising. Similar findings have been highlighted in other low-income,(45) and high-income contexts globally,(13,15,23) reiterating that lay consultation is a common process of responding to illness across different context.(46) In low resource settings of LMICs including slums, speaking to network members about illnesses or health concerns may be driven by limited access to formal health advice sources such as official symptom checkers or government health websites.(47) Reliance on informal sources of health for advice and support is common in slums, and it is linked to shortage of public healthcare facilities and inadequate capacity to pay for the private healthcare.(48)

Small lay consultation networks dominated by family members have been found in non-slum settings of LMICs and high-income settings.(17,21) Health is a sensitive issue, and people are likely to speak to small number of persons who are available to them and whom they can trust to provide the support they need.(49) In slum settings, it is not unusual for people of working age to discuss their concerns with small number of persons, as they are likely to be engaged with busy work life which makes networking challenging.(25) Keeping small networks of trusted persons in slums is also linked to frequent migration, low reciprocity among neighbours to avoid being overburdened by the needs of others and lack of trust.(24)

Our finding that married respondents were likely to speak to a lay consultant supports previous evidence that spouses are important contacts in people's treatment pathway.(19,50) Respondents who thought a health concern affected their daily activities were likely to speak to a consultant. This is in line with published evidence that people are likely to initiate treatment-seeking decisions, part of which is speaking to lay consultants, for symptoms that affect their vocational and social activities.(51) Absence from work have particularly undesirable for slum dwellers who engage in daily informal labour for a daily wage,(5) which makes it unsurprising that they engaged in lay consultation about illness concerns they perceived to affect their daily activities. As reported in other studies,(17,52) older persons were less likely to have non-family only networks or mixed networks. This relates to the socioemotional selectivity theory that an increase in age is associated with smaller social network sizes and network members that provide emotional support.(53)

For peer review only - http://bmjopen.bmj.com/site/about/guidelines.xhtml

Although our respondent's lay consultation networks were dominated by family members, they were more likely to specifically seek advice from non-family members than family members. This supports the functional specificity theory,(54) which suggests that people seek specific forms of support from their different networks. Our findings showed that non-family members were more likely than family members to provide information, while family members were more likely than non-family to provide instrumental support. Similarly, other studies suggest that friends and networks beyond one's immediate household are more likely to be sources of new information and ideas and family members are more likely to give hands-on support.(55, 56) Our findings revealed respondents obtained appraisal of symptoms and emotional support from slightly more non-family members than family members. This is line with published evidence that show that peers are important for evaluating and guiding others with similar symptoms and providing emotional support.(23, 57)

Our finding that participants that interacted with non-kin ties and extensive network members (combination of household/neighbourhood/distant network members) were significantly more likely to use informal healthcare rather than formal healthcare is similar to what has been found in other non-slum contexts in a high-income country.(58) Patent Medicine Vendors (also known as Chemists) were the most commonly used informal healthcare among our study participants, corroborating findings from other similar studies.(35,48) While information and suggestions about medicines and remedies can be retrieved from family members, non-kin ties and extensive networks can provide information about new and unfamiliar options.(58)

Our survey found that none of the participants utilised online sources in their recent illness/health concern experience and that online sources were scarcely used overall. This contrasts with findings from research conducted in high-income countries which indicated that internet sources were commonly incorporated into people's lay consultation network.(9,59,60) The contrast might be due to the digital and literacy gaps among slum dwellers in LMICs.(28)

Moreover, the survey findings highlight the concept of network homophily, whereby people tend to associate with those with whom they share similar characteristics.(61) We found that both participants and their network members had limited internet access. In low resource settings, individuals within networks face similar constraints which affects how they can support each other or share resources.(62) This reinforces the social exclusion and marginalisation experienced by people living in slum environments.

For peer review only - http://bmjopen.bmj.com/site/about/guidelines.xhtml

For peer review only

This survey had some strengths and limitations. Our survey was conducted among adults in slum settings, which are hard-to-reach communities for researchers.(24) We used a robust sampling approach, which allowed us to capture data from a representative sample of adults from 18-64 years. We asked about use of both online and personal network members to gain a holistic view of people's lay consultation network in the digital era.

This study inherited the limitations of self-reported egocentric network studies in which the data supplied by respondents were not independently verified.(63) For instance, while respondents reported that a network member did not have access to the internet, the data was not verified by interviewing the network member. However, participants' perception of the characteristics of their lay consultants is an important factor for understanding why people engage in lay consultation.(14) There may have been recall bias as participants were asked to recall information about their illness concerns and use of networks, however the short recall period minimises this risk.

In summary, people living in urban slums interact with their informal network members to make treatment-seeking decisions. During history taking, healthcare practitioners can investigate advice that people have received and tried, to understand why patients seek their help and other expectations.(64) Health programmes (such as health campaigns) in urban slums should consider engaging community members so, when consulted within their networks, they are able to deliver reliable information about health and treatment-seeking. The findings from this study are likely to be transferable to other slums of LMICs with similar socioeconomic characteristics, limited access to comprehensive and quality formal care, and dominance of informal healthcare.(4,5,48)

Contributorship statement CO conducted the data analysis and wrote the entire article. JH and FG were involved in the design of the research, reviewed and revised the paper. All authors approved the final version of the paper.

Competing interest None

Funding

This research was funded by the National Institute for Health Research (NIHR) Global Health Research Unit on Improving Health in Slums using UK aid from the UK Government to support global health research. The views expressed in this publication are those of the

For peer review only - http://bmjopen.bmj.com/site/about/guidelines.xhtml

author(s) and not necessarily those of the NIHR or the UK Department of Health and Social Care. Grant reference number 16/136/87.

Data availability statement

Data are available upon reasonable request. The data are available from the study authors on request.

Ethics approval

All participants provided informed consent to participate before taking part in the study. Ethical approval was obtained from the Biomedical and Scientific Research Ethics Sub-Committee, University of Warwick, UK (BSREC 17/19-20 AM01) and the Research Ethics Committee of the Oyo State Ministry of Health, Nigeria (AD13/479/2035).

For peer review only

References

1. UN Habitat. World Cities Report 2020: The value of sustainable urbanization. 2020.
2. UN Habitat. World Cities Report 2016: Urbanization and Development–Emerging Futures. Publisher: UN-Habitat. 2016.
3. Sunmola A, Alarape AI, Olusegun MA. The influence of house-ownership and the moderating effect of neighbourhood on life satisfaction among residents in Ibadan Metropolis, Nigeria. IFE PsychologIA: An International Journal. 2016;24(1):88-96.
4. Lilford RJ, Oyebode O, Satterthwaite D, Melendez-Torres G, Chen Y-F, Mberu B, et al. Improving the health and welfare of people who live in slums. The lancet. 2017;389(10068):559-70.
5. van der Heijden J, Gray N, Stringer B, Rahman A, Akhter S, Kalon S, et al. 'Working to stay healthy', health-seeking behaviour in Bangladesh's urban slums: a qualitative study. BMC Public Health. 2019;19(1):1-13.
6. Alaazi DA, Menon D, Stafinski T. Health, quality of life, and wellbeing of older slum dwellers in sub-Saharan Africa: A scoping review. Global Public Health. 2021;16(12):1870-88.
7. Mpanje D, Gibbons P, McDermott R. Social capital in vulnerable urban settings: an analytical framework. Journal of International Humanitarian Action. 2018;3(1):1-14.
8. Reeder KM, Sims JL, Shetty SS. Lay consultations in symptom self-care: a concept analysis for theoretical expansion in research and practice. Heart & Lung: The Journal of Cardiopulmonary and Acute Care. 2015;44(6):548.
9. Loignon C, Gottin T, Rahem N, Lafrenière D, Turcotte E, El Sherif R, et al. Maternal Experience with Online Information on Parenting and Infant Care: Qualitative Findings from Quebec, Canada. Journal of Child and Family Studies. 2022;31(7):1798-808.
10. Furstenberg AL, Davis LJ. Lay consulation of older people. Social science & medicine. 1984;18(10):827-37.
11. Perry BL, Pescosolido BA. Functional specificity in discussion networks: The influence of general and problem-specific networks on health outcomes. Social Networks. 2010;32(4):345-57.
12. Mwaka AD, Walter FM, Scott S, Harries J, Wabinga H, Moodley J. Symptom appraisal, help-seeking and perceived barriers to healthcare seeking in Uganda: an exploratory study among women with potential symptoms of breast and cervical cancer. BMJ open. 2021;11(2):e041365.
13. Reeder KM, Sims JL, Ercole PM, Shetty SS, Wallendorf M. Lay consultations in heart failure symptom evaluation. SOJ nursing & health care. 2017;3(2).
14. Freidson E. Profession of medicine: A study of the sociology of applied knowledge: University of Chicago Press; 1988.
15. Smith D, Ruston A. 'If you feel that nobody wants you you'll withdraw into your own': Gypsies/Travellers, networks and healthcare utilisation. Sociology of Health & Illness. 2013;35(8):1196-210.
16. Keboa MT, Hovey R, Nicolau B, Esfandiari S, Carnevale F, Macdonald ME. Oral healthcare experiences of humanitarian migrants in Montreal, Canada. Canadian Journal of Public Health. 2019;110:453-61.
17. Abbott KH, Stoller EP, Rose JH. The structure and function of frail male veterans' informal networks. Journal of Aging and Health. 2007;19(5):757-77.
18. Abbott KM, Bettger JP, Hanlon A, Hirschman KB. Factors associated with health discussion network size and composition among elderly recipients of long-term services and supports. Health communication. 2012;27(8):784-93.

For peer review only - http://bmjopen.bmj.com/site/about/guidelines.xhtml

19. Perry BL, Pescosolido BA. Social network activation: the role of health discussion partners in recovery from mental illness. Social science & medicine. 2015;125:116-28.
20. Pullen E, Oser C. Disadvantaged Status and Health Matters Networks among Low-Income African American Women. Social Sciences. 2017;6(3):108.
21. Queenan J, Gottlieb B, Feldman-Stewart D, Hall S, Irish J, Groome P. Symptom appraisal, help seeking, and lay consultancy for symptoms of head and neck cancer. Psycho-oncology. 2018;27(1):286-94.
22. Schoenberg NE, Amey CH, Stoller EP, Muldoon SB. Lay referral patterns involved in cardiac treatment decision making among middle-aged and older adults. The Gerontologist. 2003;43(4):493-502.
23. Stoller EP, Wisniewski AA. The structure of lay consultation networks: managing illness in community settings. Journal of Aging and Health. 2003;15(3):482-507.
24. van de Vijver S, Oti S, Oduor C, Ezeh A, Lange J, Agyemang C, et al. Challenges of health programmes in slums. The Lancet. 2015;386(10008):2114-6.
25. Gundewar A, Chin NP. Social capital, gender, and health: an ethnographic analysis of women in a Mumbai slum. Global Health Promotion. 2020;27(4):42-9.
26. Vertigans S, Gibson N. Resilience and social cohesion through the lens of residents in a Kenyan informal settlement. Community Development Journal. 2020;55(4):624-44.
27. Hu A, Loo E, Winch PJ, Surkan PJ. Filipino women's tuberculosis care seeking experience in an urban poor setting: a socioecological perspective. Health care for women international. 2012;33(1):29-44.
28. Harris B, Ajisola M, Alam RM, Watkins JA, Arvanitis TN, Bakibinga P, et al. Mobile consulting as an option for delivering healthcare services in low-resource settings in low-and middle-income countries: A mixed-methods study. Digital Health. 2021;7:20552076211033425.
29. Das M, Angeli F, Krumeich AJ, Van Schayck OC. Patterns of illness disclosure among Indian slum dwellers: a qualitative study. BMC international health and human rights. 2018;18(1):1-17.
30. Obembe T, Fonn S. Affording unavoidable emergency surgical care–The lived experiences and payment coping strategies of households in Ibadan metropolis, Southwestern Nigeria. PloS one. 2020;15(5):e0232882.
31. Onuegbu C, Larweh M, Harlock J, Griffiths F. Systematic review of lay consultation in symptoms and illness experiences in informal urban settlements of low-income and middle-income countries. BMJ open. 2021;11(12):e050766.
32. Vlahov D. Systematic surveys in informal settlements: challenges in moving toward health equity. Journal of Urban Health. 2019;96:795-6.
33. Satterthwaite D, Sverdlik A, Brown D. Revealing and responding to multiple health risks in informal settlements in sub-Saharan African cities. Journal of urban health. 2019;96:112-22.
34. Improving Health in Slums Collaborative. A protocol for a multi-site, spatially-referenced household survey in slum settings: methods for access, sampling frame construction, sampling, and field data collection. BMC Medical Research Methodology. 2019;19:1-8.
35. Fayehun O, Ajisola M, Uthman O, Oyebode O, Oladejo A, Owoaje E, et al. A contextual exploration of healthcare service use in urban slums in Nigeria. Plos one. 2022;17(2):e0264725.
36. Perry BL, Pescosolido BA, Borgatti SP. Egocentric network analysis: Foundations, methods, and models: Cambridge university press; 2018.

For peer review only - http://bmjopen.bmj.com/site/about/guidelines.xhtml

37. Onuegbu C, Harlock J, Griffiths F. How and why do people interact with lay consultants during illnesses? Qualitative study of lay consultation experiences for treatment decisions in slums of Nigeria. 2022.
38. Taiwo OJ. Modelling the spatiotemporal patterns of urban sprawl in Ibadan metropolis between 1984 and 2013 in Nigeria. Modeling Earth Systems and Environment. 2022;8(1):121-40.
39. Tella A, Balogun A-L. Ensemble fuzzy MCDM for spatial assessment of flood susceptibility in Ibadan, Nigeria. Natural Hazards. 2020;104(3):2277-306.
40. Alabi M, Babalola T, Popoola A. Tenure insecurity and incremental housing development in the peri-urban interface of Ibadan, Nigeria. Cidades Comunidades e Territórios. 2021(43).
41. Adewale BA, Ibem EO, Amole SA, Adeboye AB. Place attachment in Nigerian urban slums: Evidence from inner-city Ibadan. Cities. 2020;107:102902.
42. National Population Commission. Nigeria Demographic and Health Survey. 2018. file:///C:/Users/u2070294/Downloads/NDHS%202018%20report.pdf. Accessed June 2020

43. Fox S, Duggan M. Health online 2013. Health. 2013;2013:1-55.
44. Improving Health in Slums Collaborative. Inequity of healthcare access and use and catastrophic health spending in slum communities: a retrospective, cross-sectional survey in four countries. BMJ Global Health. 2021;6(11):e007265.
45. Esther N, Julius S, Deogratius MA. Understanding health-seeking and adherence to treatment by patients with esophageal cancer at the Uganda cancer Institute: a qualitative study. BMC Health Services Research. 2021;21(1):1-14.
46. Amzat J, Razum O, Amzat J, Razum O. Health Behaviour and Illness Behaviour. Medical Sociology in Africa. 2014:39-59.
47. Abdulai A-F, Tiffere A-H, Adam F, Kabanunye MM. COVID-19 information-related digital literacy among online health consumers in a low-income country. International journal of medical informatics. 2021;145:104322.
48. Watson S. Pharmacies in informal settlements: a retrospective, cross-sectional household and health facility survey in four countries. BMC health services research. 2021;21:1-10.
49. Amoah PA, Edusei J, Amuzu D. Social networks and health: understanding the nuances of healthcare access between urban and rural populations. International journal of environmental research and public health. 2018;15(5):973.
50. Cooper S, Gilbert L. The role of 'social support'in the experience of fibromyalgia–narratives from S outh A frica. Health & social care in the community. 2017;25(3):1021-30.
51. Zola IK. Pathways to the doctor—from person to patient. Social Science & Medicine (1967). 1973;7(9):677-89.
52. Cook-Craig P, Ely G, Flaherty C, Dignan M, White CR. Seeking health advice from social networks in low-income urban neighborhoods. American journal of health behavior. 2012;36(6):723-35.
53. Lang FR, Staudinger UM, Carstensen LL. Perspectives on socioemotional selectivity in late life: How personality and social context do (and do not) make a difference. The Journals of Gerontology Series B: Psychological Sciences and Social Sciences. 1998;53(1):P21-P30.
54. Wellman B, Wortley S. Different strokes from different folks: Community ties and social support. American journal of Sociology. 1990;96(3):558-88.
55. Granovetter MS. The strength of weak ties. American journal of sociology. 1973;78(6):1360-80.

For peer review only - http://bmjopen.bmj.com/site/about/guidelines.xhtml

56. Roberts SG, Dunbar RI. The costs of family and friends: an 18-month longitudinal study of relationship maintenance and decay. Evolution and Human Behavior. 2011;32(3):186-97.
57. Hood S, Golembiewski E, Benbow K, Sow H, Sanders Thompson V. Who can I turn to? Emotional support availability in African American social networks. Social Sciences. 2017;6(3):104.
58. Schafer MH. Discussion networks, physician visits, and non-conventional medicine: probing the relational correlates of health care utilization. Social Science & Medicine. 2013;87:176-84.
59. Nettleton S, Hanlon G. 'Pathways to the Doctor'in the Information Age: the Role of ICTs in Contemporary Lay Referral Systems. New technologies in health care: Challenge, change and innovation. 2006:57-70.
60. Sinha A, Porter T, Wilson A. The use of online health forums by patients with chronic cough: qualitative study. Journal of Medical Internet Research. 2018;20(1):e19.
61. Flatt MJD, Agimi MY, Albert SM. Homophily and health behavior in social networks of older adults. Family & community health. 2012;35(4):312.
62. Lubbers MJ, Small ML, García HV. Do networks help people to manage poverty? Perspectives from the field. The ANNALS of the American Academy of Political and Social Science. 2020;689(1):7-25.
63. O'malley AJ, Arbesman S, Steiger DM, Fowler JH, Christakis NA. Egocentric social network structure, health, and pro-social behaviors in a national panel study of Americans. PloS one. 2012;7(5):e36250.
64. Hiscock J. Informal interactions about health: connectedness, surveillance and the construction of a moral identity: The University of Manchester (United Kingdom); 2013.

For peer review only

For peer review only - http://bmjopen.bmj.com/site/about/guidelines.xhtml

Table 1: Survey instrument

		**Section One: Sociodemographic Characteristics of respondents**<br><br>I would like to begin by asking you some questions about yourself.	
1	Age	How old were you on your last birthday?	(____)
2	Gender	What is your sex?	Male………..1<br>Female……..2
3	Digital Communication	a. Do you carry a mobile phone day-to-day?	Yes………………1<br>No………………...2<br>Don't know………3
		b. How many days in the week do you have access to internet/Wi-Fi (for example, Facebook, email) using digital communication device (like smart phone, laptop, tablet)?	Everyday…………..1<br>Almost every day (more than 21 days a month but not every day)……2<br>About five times a week……..3<br>About three times a week……..4<br>Once in a week……5<br>Never………………6

Section 2: Structure of Lay Consultation Now, I want to ask you about how you discuss your health concerns or symptoms with members of your social networks, that is people who you are connected or related to, before seeking care or treatment.

S/n	Questions	Response categories
4	When did you last have an illness or any health concern that concerned you enough that you felt you needed some form of care or advice?	
5	What was the symptom or health concern?	
6	How did this symptom/health concern affect your daily functioning/activities?	
7	Did you discuss the symptom or health concern with anyone you are related or connected to, whether online or offline, or any online platform?	Yes ………………………1<br>No……………………….2
9	If yes, who or which platform did you first discuss the experience with?	a.
10	In all, how many people did you talk to?	
11	Did you discuss the illness/ health concern with anyone else or on any other platform? (asides the person you named in question 9) Please list them in the order in which you interacted with them	
		b
		c
		d
		e
		f
		g
		h
		i
12. Please, tell me more about each of the persons that you discussed with during this illness episode		

For peer review only

What is the person's initials/Nick Name	Which of the following best describes your relationship to (Name)?<br>Spouse (1)<br>Ex-spouse (2)<br>Friend (3)<br>Neighbor (4)<br>Romantic/sexual partner (5)<br>Co-worker or boss (6)<br>Parent (7)<br>Mother-in-law (8)<br>Father-in-law (9)<br>Child (10)<br>Brother or sister (11)<br>Other relative of yours (12)<br>Minister, priest or other clergy (13)<br>Medical Dr (14)<br>Community Health Worker (15)<br>Other (SPEC) (16)	Is (Name) male or female?<br><br>Male = 1<br>Female =2	How did you reach (Name)?<br><br>Face to face =1<br>Chat =2<br>Text message =3<br>E-mail = 4<br>Video call =5

How close do you live to (Name)?	What kind of medicine does (Name) Advocate?	Does (Name) have access to the internet?	Why did you discuss your symptom or health concern with (NAME)?	Which of the following was the main support you received from (NAME) for this illness episode?
Same household = 1<br>Same compound = 2<br>Same street = 4<br>Same LCDA = 5<br>Same state =6<br>Other (specify) = 7	Modern = 1<br>Traditional =2<br>Combination of Both =3<br>Others (specify) = 4	Yes =1<br>No =2<br>I don't know =3	To obtain information on the cause(s) of the symptom/health problem = 1<br><br>To ask for advice about formal health care provider = 2<br><br>To ask where to seek alternative care = 3<br><br>To learn about home remedies = 4<br><br>To report the illness =5<br><br>Because I am expected to tell (NAME) about my health condition?<br><br>Others (specify)------	Information on the causes/health problem (1)<br><br>Advice on where to seek care (2)<br><br>Information about formal health provider(4)<br><br>Information about alternative health provider (5)<br><br>Others (specify)

For peer review only - http://bmjopen.bmj.com/site/about/guidelines.xhtml

13	Did you seek care/treatment for the health concern?	Yes<br>No (if no, why?)
14	Which service/option did you use?	Medical doctor (including surgeon, gynaecologist, psychiatrist, ophthalmologist)………………………...1<br>Registered Nurse………………………………2<br>Auxiliary nurse…………………………………3<br>Midwife………………………………4<br>Dentist…………………………………5<br>Physiotherapist or chiropractor ………………6.<br>Traditional medicine practitioner ………………………7<br>Pharmacist, druggist……………………...8<br>Home remedy…………………………..9<br>Spiritualist………………………………10<br>Self-medication………………………………11<br>Other, please specify…………………………..12
**Section 3: Use of Digital Communication for lay consultation**<br>Now, I want to ask about how you use online platforms or forums to discuss your symptoms or health concern that you feel requires some form of care.		
15	Do you use online platform(s) to discuss your symptoms or health concern that you feel requires some form of care?	Yes…………………………………1<br>No……………………………………..2 (
16	If yes, which do you use?	(pick as many that applies to you)<br><br>Google………………………………………1<br>Bing……………………………………………2<br>Yahoo…………………………………………..3<br>WebMD………………………………………4<br>Wikipedia……………………………………5<br>Facebook……………………………………6<br>You-tube……………………………………..7<br>Others, Please specify____________
17	In what ways do you share such health concerns/symptoms on online platforms?	Browsing……………………………1<br>Posting health related questions on health professional sites or social media platforms…2<br>Posting health related questions on platforms with persons personally known to you….3<br>Posting health related questions on sites/platforms with more general audience…3<br>Others (specify)……..
18	What do you post or share online?	Specific question about your health…………1<br>Comment……………………………………2<br>Story about your personal health experience...3<br>Other specify ______________________
**Section 4: Further Socio-demographic Characteristics of Respondents**<br>In this last part, I want to ask some further questions about yourself		

For peer review only

19	Employment	As you know, some people take up jobs for which they are paid in cash or kind. Others sell things, have a small business or work on the family farm or in the family business.<br>In the last seven days, have you done any of these things or any other work or have a business which you were absent from in the last seven days?	Yes..………….……..1<br><br>No……………………2
20	Marital status	What is your current marital status?	Married or living together……………1<br>Divorced / separated…………….2<br>Widowed………..…3<br>Never-married and Never lived together…….4
21	Medical Insurance	Are you under any kind of medical insurance?	Yes…………………..1<br>No……………………2
22	Migration status	Did you always live in this neighborhood?<br><br>(this question asks if the respondent was born here)	Yes…………..1<br>No……………2

Table 2: Nature of illness or health concerns and perceived effects of illness or health concern on daily activities

Nature of illness or health concern		
**Infectious symptoms**		
Malaria	126	26.3
Malaria with headache/weakness/nausea/runny nose/ache	37	6.7
Typhoid	10	2.1
Measles-like rash	7	1.5
Malaria and typhoid	7	1.5
Malaria and ulcer	4	0.8
Typhoid and cough	1	0.2
Diarrhoea	2	0.4
Headache, cold and weakness	2	0.4
	196	40.8
**Headaches**		
Headache	55	11.5
Headache and body pain	5	1
Headache and weakness	3	0.8
Headache and fever	4	0.8
Headache and body ache	3	0.6
Headache and cold	1	0.2
Headache and catarrh	1	0.2
	72	15.0
**General pain and weakness**		
Body aches/body pain	34	6.3
Body weakness	25	5.2

For peer review only - http://bmjopen.bmj.com/site/about/guidelines.xhtml

Shortage of blood	1	0.2
Body pain and weakness	2	0.4
Tiredness and temperature	1	0.2
Weakness and dizziness	2	0.4
	65	13.5
**Specific musculoskeletal pain**		
Leg pain/arm pain/shoulder/waist/thighs	29	4.4
Joint pain/knee pain	12	2.5
Chest pain	4	0.6
Back pain/ upper/lower	4	0.4
	49	10.2
**Gastrointestinal and abdominal issues**		
Piles	5	1
Stomach pain/lower abdominal pain/diarrhoea	17	3.5
Stomach ulcer	2	0.2
Stomach pain and headache	5	1
	29	6.0
**Others**		
Cough	5	1
Fever/high temperature	17	3.5
Accident/injuries/bruises	3	2.3
Catarrh	8	1.5
Menstrual cramps	3	0.8
Eye pain	4	0.8
Toothache	4	0.8
Pregnancy symptoms	4	0.8
Cough and catarrh	3	0.6
Anxiety/depression/sadness	3	0.6
Cataract/cloudy vision	3	0.6
High blood pressure	2	0.4
Miscarriage	2	0.4
Stress	2	0.4
Arthritis	2	0.4
Mouth ulcer	1	0.2
Dry throat	1	0.2
Vagina bleeding following menstruation	1	0.2
Fever and high bp	1	0.2
Fever, pile, and dizziness	1	0.2
Hernia surgery	1	0.2

Stroke	1	0.2
Hypertension	1	0.2
Stiffness	1	0.2
Smelly burps	1	0.2
	69	14.4
Missing	1	0.2
**Perceived effect of illness/health concern on daily activities**	**N=480**	**%**
Some effects	266	55.4
No effects	214	44.6
**Kinds of effects**	**N=266**	**%**
Unable to go to work	82	30.8
Unable to perform normal household chores	59	22.2
Unable to work full time	30	11.3
Could not do any of my normal activities	39	14.7
Was on bed rest	24	9.1
Unable to perform religious activities	12	4.5
Could not exercise	8	3
Unable to sleep normally	4	1.5
Affected work and socialisation	4	1.5
Unable to socialise with friends	2	0.8
Hospitalised	1	0.4
Could not eat	1	0.4

Table 1. Association between relationship with network members and the reason for which they were consulted

		**Reason for consultation**		**Chi-square**	**DF**	**P-value**
		Consulting	Non-consulting			
Relationship	Family	39 (7.4%)	490 (92.6%)	50.781	1	0.000
	Non-family	45 (29.2%)	109 (70.8%)			

Table 2. Association between relationship with network members and support provided by network members

		**Forms of support**				**X2**	**DF**	**P-value**
		**Information**	**Instrumental support**	**Emotional support**	**Appraisal**			

For peer review only - http://bmjopen.bmj.com/site/about/guidelines.xhtml

Relationship	Family	198 (37.4%)	276 (52.2%)	31 (5.9%)	24 (4.5%)	16.499	3	0.001
	Non-family	79 (52.3%)	51 (33.8%)	10 (6.6%)	11 (7.3%)			

For peer review only

STROBE 2007 (v4) Statement—Checklist of items that should be included in reports of *cross-sectional studies*

Section/Topic	Item #	Recommendation	Reported on page #
**Title and abstract**	1	(*a*) Indicate the study's design with a commonly used term in the title or the abstract	1
		(*b*) Provide in the abstract an informative and balanced summary of what was done and what was found	2
**Introduction**			
Background/rationale	2	Explain the scientific background and rationale for the investigation being reported	3-4
Objectives	3	State specific objectives, including any prespecified hypotheses	4
**Methods**			
Study design	4	Present key elements of study design early in the paper	5
Setting	5	Describe the setting, locations, and relevant dates, including periods of recruitment, exposure, follow-up, and data collection	5-6
Participants	6	(*a*) Give the eligibility criteria, and the sources and methods of selection of participants	6
Variables	7	Clearly define all outcomes, exposures, predictors, potential confounders, and effect modifiers. Give diagnostic criteria, if applicable	7-8
Data sources/ measurement	8*	For each variable of interest, give sources of data and details of methods of assessment (measurement). Describe comparability of assessment methods if there is more than one group	7-8
Bias	9	Describe any efforts to address potential sources of bias	Not applicable
Study size	10	Explain how the study size was arrived at	6
Quantitative variables	11	Explain how quantitative variables were handled in the analyses. If applicable, describe which groupings were chosen and why	8
Statistical methods	12	(*a*) Describe all statistical methods, including those used to control for confounding	8
		(*b*) Describe any methods used to examine subgroups and interactions	
		(*c*) Explain how missing data were addressed	
		(*d*) If applicable, describe analytical methods taking account of sampling strategy	
		(*e*) Describe any sensitivity analyses	
**Results**			

For peer review only

Participants	13*	(a) Report numbers of individuals at each stage of study—eg numbers potentially eligible, examined for eligibility, confirmed eligible, included in the study, completing follow-up, and analysed	9
		(b) Give reasons for non-participation at each stage	9
		(c) Consider use of a flow diagram	
Descriptive data	14*	(a) Give characteristics of study participants (eg demographic, clinical, social) and information on exposures and potential confounders	9-10
		(b) Indicate number of participants with missing data for each variable of interest	9
Outcome data	15*	Report numbers of outcome events or summary measures	
Main results	16	(*a*) Give unadjusted estimates and, if applicable, confounder-adjusted estimates and their precision (eg, 95% confidence interval). Make clear which confounders were adjusted for and why they were included	11-16
		(*b*) Report category boundaries when continuous variables were categorized	
		(*c*) If relevant, consider translating estimates of relative risk into absolute risk for a meaningful time period	
Other analyses	17	Report other analyses done—eg analyses of subgroups and interactions, and sensitivity analyses	
**Discussion**			
Key results	18	Summarise key results with reference to study objectives	17-20
Limitations	19	Discuss limitations of the study, taking into account sources of potential bias or imprecision. Discuss both direction and magnitude of any potential bias	19
Interpretation	20	Give a cautious overall interpretation of results considering objectives, limitations, multiplicity of analyses, results from similar studies, and other relevant evidence	19-20
Generalisability	21	Discuss the generalisability (external validity) of the study results	20
**Other information**			
Funding	22	Give the source of funding and the role of the funders for the present study and, if applicable, for the original study on which the present article is based	20

*Give information separately for cases and controls in case-control studies and, if applicable, for exposed and unexposed groups in cohort and cross-sectional studies.

Note: An Explanation and Elaboration article discusses each checklist item and gives methodological background and published examples of transparent reporting. The STROBE checklist is best used in conjunction with this article (freely available on the Web sites of PLoS Medicine at http://www.plosmedicine.org/, Annals of Internal Medicine at http://www.annals.org/, and Epidemiology at http://www.epidem.com/). Information on the STROBE Initiative is available at www.strobe-statement.org.

For peer review only
